# Response diversity increases functional but decreases compositional stability of grassland communities

**DOI:** 10.1111/nph.71546

**Published:** 2026-09-06

**Authors:** Manuel Pitzer, Vincent Zieschank, Wolfgang Trutschnig, Robert R. Junker

**Affiliations:** ^1^ Marburg University, Department of Biology, Evolutionary Ecology of Plants Marburg 35043 Germany; ^2^ Research Group for Data Science, Statistics, Stochastics, Department for Artificial Intelligence & Human Interfaces University of Salzburg Salzburg A 5020 Austria

**Keywords:** biodiversity, compositional stability, ecosystem stability, functional stability, grassland, land use, plant community, response diversity

## Abstract

- The insurance hypothesis of biodiversity assumes that biodiversity increases ecosystem stability when functionally redundant species exhibit distinct responses to environmental changes, allowing some species to compensate for the loss of others. However, empirical evidence remains scarce.
- Using a novel approach to quantify the responses of 96 plant species to land use, we tested the hypothesis combining field data with a common garden experiment. By subjecting transplanted grassland plant communities to different land‐use intensities, we quantified response diversity (defined as the variability of responses among species to the same environmental factor), species composition, and functionality from spectral data before and after experimental changes.
- Our results show that land‐use intensification reduced response diversity of plant communities in the field and the common garden. Moreover, our results indicate that response diversity increases functional stability but decreases stability in terms of species composition (temporal β‐diversity), that is, response‐diverse communities tended to lose species with fewer replacements while response‐depleted communities mainly experienced species turnover.
- Our work highlights that response diversity underlies multiple dimensions of ecosystem stability as it stabilizes functionality but destabilizes species composition. We further discuss future research directions to adapt the concept of response diversity under global change scenarios.

## Graphical abstract

*Response space* of grassland plant species to increasing land‐use intensity based on *response dissimilarities* quantified by using a novel approach. The *response space* clusters species that benefit from land‐use intensification (‘*winners*’, green), are negatively affected (‘*losers*’, yellow), and those with nonmonotonic responses (blue). Our results support the insurance hypothesis of biodiversity: response diversity increases functional stability but decreases stability in terms of species composition.

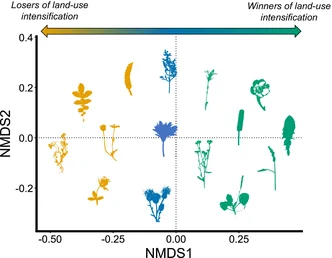

## Introduction

Responses of ecological communities to anthropogenic perturbations define ecosystem stability and thus long‐term functionality (Hautier *et al*., 2015; Blüthgen *et al*., 2016; Wagg *et al*., 2022). It is well established that biodiversity plays a crucial role in enhancing communities' resistance and resilience to global change, thereby supporting the maintenance of ecosystem functions (Tilman *et al*., 2006; Hautier *et al*., 2015; Wang *et al*., 2019). The insurance hypothesis of biodiversity, as one potential mechanism underlying the link between biodiversity, stability, and function (Yachi & Loreau, 1999; Mori *et al*., 2013), proposes that in biodiverse communities some species may functionally compensate the loss of other species and thus prevent ecosystem degradation (Díaz & Cabido, 2001; Lisner *et al*., 2023; Bazzichetto *et al*., 2024). A prerequisite for this compensation is heterogeneous responses (e.g. in abundance or performance) to environmental changes of functionally redundant species within a community (Mori *et al*., 2013; Nash *et al*., 2016). Accordingly, *‘response diversity’*, defined as interspecific differences in responses to abiotic factors within functionally redundant species, has been recognized as an important component of biodiversity that promotes ecosystem stability (Sasaki *et al*., 2019; Ross & Sasaki, 2024).

Ecosystem stability is a multidimensional property that quantifies the temporal constancy and resistance of species diversity and composition or of ecosystem functions under fluctuating or shifting environmental conditions over time (Donohue *et al*., 2013, 2016; Kéfi *et al*., 2019). Accordingly, effects of environmental changes and disturbances such as land‐use intensification may include alterations in the α‐diversity of communities and changes in species composition due to immigration and extinction of species (known as compositional turnover, β‐diversity; Pimm, 1984; Donohue *et al*., 2013, 2016). Additionally, environmental changes may also affect the functioning of ecosystems or proxies thereof like spectral indices based on vegetation reflectance (e.g. Normalized Difference Vegetation Index (NDVI); see Pettorelli, 2013; Mazzochini *et al*., 2024; Varouchakis *et al*., 2025). As diversity components as well as functioning result from the performance of individual species (Elmqvist *et al*., 2003; Loreau & De Mazancourt, 2013; Laughlin, 2014), response diversity, quantifying the variability in responses to environmental change in functionally redundant species within communities, may represent an underlying mechanism of the biodiversity–stability relationship (Van Ruijven & Berendse, 2010; Bhaskar *et al*., 2018).

While the potential of response diversity in explaining the relation between biodiversity and ecosystem stability is widely accepted (McCann, 2000; Blüthgen *et al*., 2016; Schnabel *et al*., 2021; Polazzo *et al*., 2024; Nonaka & Sasaki, 2025), the observational, experimental, and statistical methods required to quantify response diversity have not converged to best practices so far (Ross *et al*., 2023; Ross & Sasaki, 2024). For instance, the use of response trait diversity as proxy for response diversity (Craven *et al*., 2016; Aquilué *et al*., 2020) has been criticized for the difficulty in discriminating response from effect traits leading to uncertainties in the interpretation of results (Mori *et al*., 2013; Ross *et al*., 2023). More promising may be ‘*performance–environment relationships*’ (Ross *et al*., 2023) that directly reflect species responses to environmental changes. Based on performance proxies (such as abundance in studies on pollinators; Winfree & Kremen, 2009; Stavert *et al*., 2017) or the intrinsic rate of increase in studies on protists and plants (Leary & Petchey, 2009; McCann, 2016; Ross *et al*., 2023) of several species along the same environmental gradient, previous studies frequently considered the interaction term ‘*species × environment*’ as indicative of response diversity. Alternatively, direct and pairwise comparison of species' performance curves is a promising approach that reveals more detailed information on species' responses when using abundances as performance proxy along environmental gradients (Fig. 1a; see Ross *et al*., 2023; see also Stavert *et al*., 2017; Ruiz‐Moreno *et al*., 2024).

**Fig. 1:**
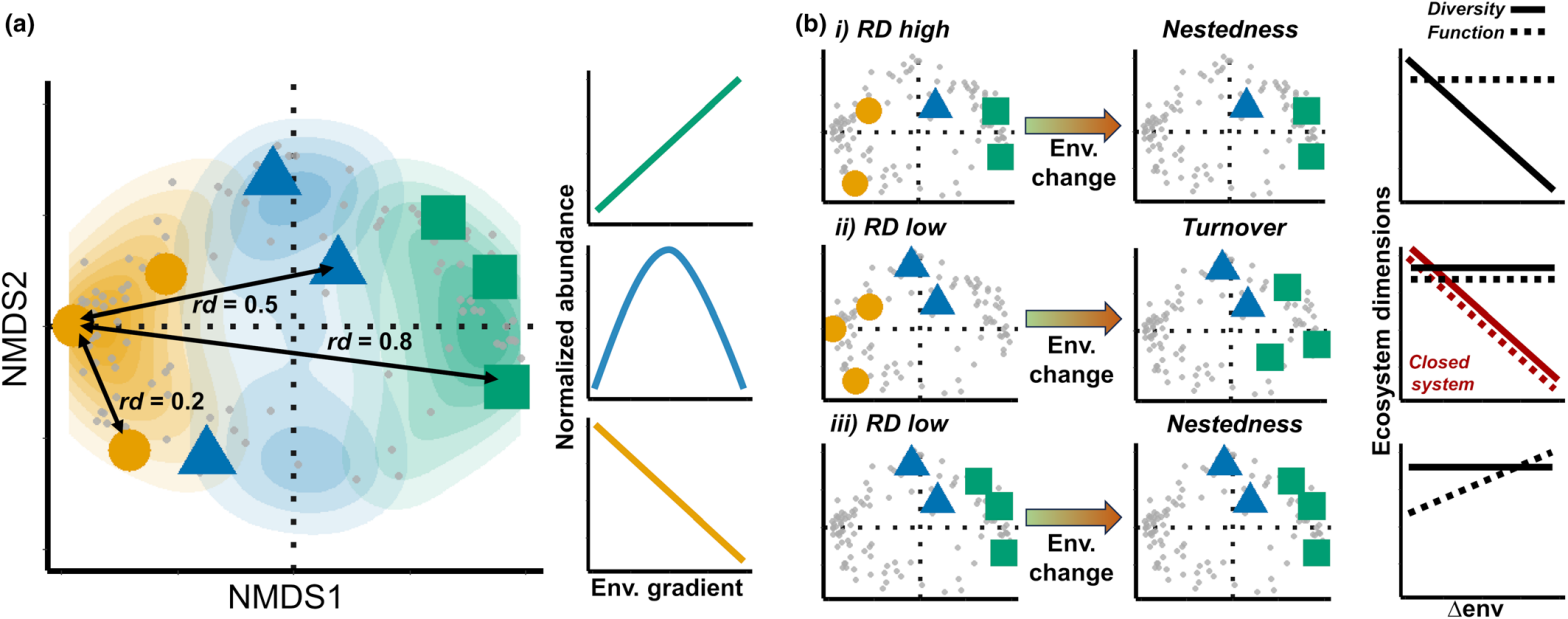
Expected species and community responses to changing environments in relation to response dissimilarity and diversity. Changing environments may include global change components such as warmer temperatures or increasing land use, or natural gradients such as elevation, succession, or latitude. (a) An ordination (*response space*) based on the distances between species‐specific abundance–environment curves (such as seen on the right side) of *n* = 9 species, which are described by two empirical checkerboard copulas (response dissimilarity *rd* $\in \left[0,1\right]$). The closer two species are positioned, the more similar they respond to the same environmental gradient. Actual responses are defined as normalized, species‐specific abundance–environment curves and are assigned to negative (yellow circles), positive (green squares), and nonmonotonic responses (blue triangles). Smaller, gray dots represent species with differing responses to the same gradient, which are not present in the community but in the regional species pool. Density distributions are based on the types of responses of the regional species pool. (b) Expected community responses to changing environments in terms of species composition and diversity (solid lines) as well as ecosystem function (dashed lines) dependent on initial response diversity *RD*. Initial communities (i, ii, iii) consist of five species that respond to an environmental gradient or change and differences (distances) in those responses contribute to communities´ response diversity (here, depicted as specific response spaces, see (a)). Thus, communities consisting of species covering the whole response gradient from negative over nonmonotonic to positive responses feature a high(er) response diversity (i), whereas communities with species covering a section of the response gradient only feature a low(er) response diversity (ii, iii). After positive changes (increases) in an environmental condition (indicated by the arrow and ‘∆env’, i.e. after climate warming or increased land‐use intensity), we expect the following consequences. (i) Community with high response diversity: species that positively respond to positive changes in the environment will dominate the community, which will lead to a reduced species diversity but a constant ecosystem function as predicted by the insurance hypothesis of biodiversity (Yachi & Loreau, 1999). The new community will have a reduced response and species diversity and species of the new community will be nested in the species of the previous community. (ii) Communities with low response diversity and species with on average negative responses to positive environmental changes: Negatively responding species will suffer and possibly go locally extinct leading to a decrease in species and response diversity as well as a decreased ecosystem function without immigration, that is in a closed system (red lines). Species immigrating from the regional species pool, that is positively responding species (former gray dots), may compensate decreases in species diversity as well as losses in functionality (black lines). In these cases, we would expect a turnover in species composition. (iii) Communities with low response diversity and species with on average positive responses to positive environmental changes: Positively responding species will retain species diversity and (low) response diversity but may even increase their functionality. The new species composition will be nested in the previous species composition.

Ongoing environmental changes such as climate change or land‐use intensification are characterized by unidirectional shifts in mean conditions. Whereas Yachi and Loreau assumed environmental fluctuations (positive and negative deviations from a constant mean) in their original work (Yachi & Loreau, 1999), more recent syntheses now treat the insurance hypothesis and response diversity as intrinsically linked concepts applicable to both stochastic fluctuations and directional environmental shifts (Elmqvist *et al*., 2003; Mori *et al*., 2013; Ross & Sasaki, 2024). These unidirectional environmental changes are, however, often also accompanied by changes in the magnitude and frequency of fluctuations, which prevents a clear separation of both effects. Moreover, Yachi and Loreau assumed a closed system, whereas natural communities represent open systems allowing for dynamic changes in species abundances and processes such as immigration from regional species pools and local extinction, which may play a pronounced role in natural communities while the basic underlying theory may still hold true (Yachi & Loreau, 1999). For example, the intensification of land use is among the most severe threats for grassland ecosystems (Foley *et al*., 2005; Newbold *et al*., 2015; Díaz *et al*., 2019) and poses stress on plant communities that negatively respond to mowing and/or fertilization (Wesche *et al*., 2012; Socher *et al*., 2013; Allan *et al*., 2015). In communities consisting of species with divergent responses to land‐use change (high response diversity potentially mediating a high resistance potential to increases and decreases in land‐use intensity), those species that positively respond to land‐use change (i.e. increase in abundance; *‘winners’*) will become dominant, whereas species that negatively respond to this change (i.e. decrease in abundance; *‘losers’*) may become weak competitors and will eventually be completely outcompeted by other species better adapted to new conditions (Boulangeat *et al*., 2012; Busch *et al*., 2019). Consequently, in response‐diverse communities species diversity will likely decrease and species composition will change in a way that the new communities will be composed of the winners of the change only (i.e. a subset of species nested within the species of the previous community; Fig. 1b(i); Gossner *et al*., 2016). However, such response‐diverse communities may be more resistant and thus more stable in terms of ecosystem functioning when the remaining species compensate for the functions not provided anymore by the lost species (Walker *et al*., 1999; Chillo *et al*., 2011; Fig. 1b(i)). As an example, ecosystem productivity may be unaffected despite species decline when remaining plant species compensate the biomass production of the lost species and thus can be considered as functionally redundant to some degree (see Hooper *et al*., 2005). By contrast, communities consisting of species that collectively respond negatively to land‐use change (low response diversity potentially mediating a low resistance potential) would be expected to decline in functionality, unless the poor performance or even loss of loser species is compensated by immigrating species that thrive under the new conditions (i.e. communities characterized by strong turnover; Stavert *et al*., 2017; Fig. 1b(ii)). Solely in communities consisting of species that collectively respond positively to land‐use change, both species diversity and composition may be stable and functionality may even increase if species perform better under the new environmental conditions (see Cardinale *et al*., 2006; Cadotte, 2017; Fig. 1b(iii)). Consequently, we extended the framework of the insurance hypothesis of biodiversity (Yachi & Loreau, 1999) by allowing immigration and local extinctions in this study.

Using a novel, copula‐based approach that accounts for monotonic (constantly positive or negative slopes) and nonmonotonic relationships (with both positive and negative slopes) between plant abundance and environmental gradients (for methodological details, see Supporting Information), which are commonly found in natural systems (Hanusch *et al*., 2023), we calculated pairwise response dissimilarities *rd*_*ij*_ among *n* = 96 plant species frequently found in the plots of the Biodiversity Exploratories (Fischer *et al*., 2010). These response dissimilarities *rd*_*ij*_ were combined with a common garden experiment, which consisted of grass sods originating from a selection of the same field plots that strongly varied in land‐use intensity (measured as the land‐use intensity index LUI, Blüthgen *et al*., 2012). LUI ranged from protected areas with minimal usage to intensively managed grasslands. Each grass sod was divided into four subplots that were subjected to one of the four experimental treatments that differed in mowing frequency and amount of applied fertilizer for three growing seasons, which ultimately led to changes in LUI in all subplots. We recorded the abundance of plant species in June 2020 (before treatments started) and July 2022 (after treatment) and measured the NDVI of each community using a 3D‐plant scanner (He *et al*., 2023; Zieschank & Junker, 2023; Zieschank *et al*., 2025) in each of the subplots in late June 2020 (before treatments started) and mid‐August in 2022 (after treatment) to track compositional and functional changes due to experimental treatments and infer stability. Based on the assumptions outlined above (Fig. 1), we hypothesize that response diversity increases functional stability (due to compensation) but decreases stability in terms of species diversity and composition under unidirectional land‐use changes. More specifically, in the experimental common garden setting we tested hypotheses on the relationships between response diversity, functional stability, and temporal changes in species:

1. Species exhibit distinct responses to changes in land‐use intensity reflected in pairwise response dissimilarities *rd*_*ij*_ (Fig. 1a). Resulting from historically experienced land‐use intensities, initial communities in the common garden subplots differ in species composition and in response diversity (see Fig. 1b).
2. Land‐use intensity negatively affects response diversity both in the field and in the common garden as it favors winners only.
3. Following land‐use changes, communities featuring a high response diversity are more stable in ecosystem functioning but less stable in species diversity (Fig. 1b(i)) than communities with low response diversity since *‘loser species’* may vanish while *‘winner species’* prevent losses in functionality (Fig. 1b(ii,iii)).
4. Changes in community composition are more pronounced in response‐diverse communities than in response‐depleted communities. The nestedness component dominates temporal β‐diversity in response‐diverse communities when *‘loser species’* are lost with few or no replacements, but turnover components dominate in communities featuring a low response diversity when *‘loser species’* are replaced by immigrating *‘winner species’* (see Fig. 1b(ii)).

## Materials and Methods

### Field data

The study was conducted as part of the Biodiversity Exploratories, a large‐scale platform for functional biodiversity research. It comprises three long‐term research sites (‘Exploratories’) at three regions (Schwäbische Alb, Hainich, and Schorfheide‐Chorin) in Germany spanning a north–south gradient with *c*. 400 km distance between the sites, where biodiversity and land use have been continuously recorded on *n* = 150 grassland plots since 2006 (Fig. 2a(i,ii); Fischer *et al*., 2010). Land use includes the amount of applied fertilizer (kg N ha^−1^ yr^−1^), grazing intensity (livestock units d^−1^ ha^−1^ yr^−1^), and mowing frequency (number of cuts yr^−1^), which are commonly applied in combination (Blüthgen *et al*., 2012). Information about intensities at each plot *x* is gathered by surveying land owners (Vogt *et al*., 2019) and integrated into the land‐use intensity index LUI, which quantifies the strength in total land use, has been shown to negatively correlate with plant diversity (Blüthgen *et al*., 2012) and can be calculated for freely selectable time frames using the LUI calculation tool (Ostrowski *et al*., 2020) implemented in BExIS (doi: 10.17616/R32P9Q) provided by the Biodiversity Exploratories (see Supporting Information Methods S1, S2 for detailed calculation of LUI following Blüthgen *et al*., 2012). Grassland plots had a mean LUI of 1.79 (range: 0.58–3.25). In all 150 plots, plant species were identified on a fixed area of 4 × 4 m in May/June from 2008 to 2022 and the abundance of each plant species was estimated (cover in %; Fig. 2a(ii)). The dataset is publicly available on BExIS (https://www.bexis.uni‐jena.de/, ID #31389, v.7, Hinderling & Keller, 2023).

**Fig. 2:**
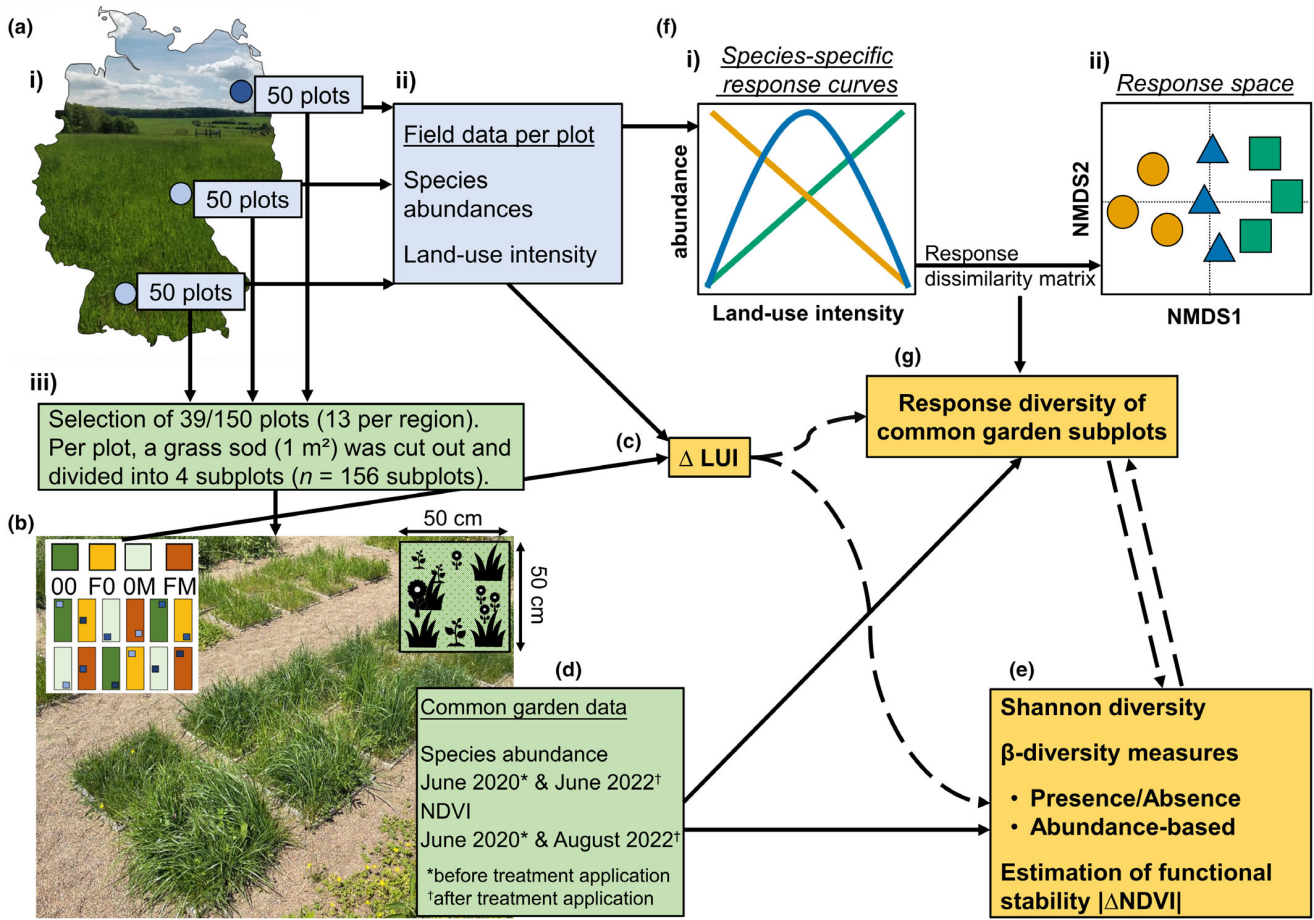
Schematic study framework and data collection. As part of the Biodiversity Exploratories (Fischer *et al*., 2010), our study combines field data (blue boxes) with a common garden experiment (green boxes) to investigate the mechanism of response diversity under land‐use change (yellow boxes) in grassland plant communities. Bold variables were used for statistical analysis. Dashed arrows indicate expected interactions and relationships. Solid arrows show the study framework, work flows and data acquisition processes. (a) (i) Map showing the locations of the three regions of the Biodiversity Exploratories (Photograph: V. Zieschank). In each region, 50 agriculturally used grassland plots are studied. (ii) For each plot, land‐use intensity, measured by the land‐use intensity index (LUI; Blüthgen *et al*., 2012) and the vegetation coverage (species abundance) have been recorded. (iii) Per region, 13 grassland plots with variable historical land‐use intensities (39/150 plots) were chosen and a grass sod of 1 m^2^ per plot was cut out and quartered (*n* = 156 subplots). (b) Each subplot was transplanted into a common garden in 2020 and assigned to one of four experimental treatments in a semi‐random block design: ‘00’ (mowing once per year), ‘0M’ (mowing twice per year), ‘F0’ (mowing once per year and fertilizing) and ‘FM’ (mowing twice per year and fertilizing) ultimately changing the LUI in each subplot (∆_LUI_, either increases or decreases; Photograph: R. R. Junker) (c). (d) In the common garden, species abundances were estimated and the Normalized Difference Vegetation Index (NDVI) of each subplot was measured in 2020 before the start of the experiment and in mid‐august 2022 with an automated 3D plant scanning system. (e) Shannon diversity, abundance‐based temporal β‐diversity (Bray–Curtis distances) and Sørensen's dissimilarity (temporal β‐diversity based on presence–absence data) were calculated based on species data from 2020 and 2022. Changes in Shannon diversity (∆_Shannon_) were determined by subtracting the values of 2020 values from those of 2022. Functional stability was estimated as the absolute changes in NDVI (|∆_NDVI_|) for each community. (f) (i) Applying a moving frame across *k* = 10 plots to the abundances of each given species, species‐specific abundance–environment relationships to land‐use intensity have been calculated as response proxies. Using empirical checkerboard copulas to describe the species‐specific abundance–environment relationships, D1‐distances (distances between two empirical checkerboard copulas) were calculated for each pair of species and multiplied by the constant 2 to yield response dissimilarities *rd*_*ij*_ $\in \left[0,1\right]$. (ii) Compiled into a response dissimilarity matrix, *rd* was used to establish a *‘response space’* by nonmetric multidimensional scaling (NMDS). In such ordinations, species are positioned according to their responses to an environmental gradient and cluster the more similar their responses are. Assignment of species responses was done by using Spearman's Rho and the index of monotonicity *m* as defined in Hanusch *et al*. (2023). (g) Based on the response dissimilarity matrix (field data) and the subplot‐specific species abundances (common garden data), functional dispersion was used to obtain response diversity *RD* for each subplot in 2020 and 2022. Changes in response diversity were calculated by subtracting the response diversity of 2020 from the response diversity of 2022 (∆_RD_).

### Common garden

Across the Exploratories, we selected *n* = 13 plots per region that covered a large range of mean land‐use intensities (2006–2019) and collected grass sods with a size of 1 m^2^ and 10 cm depth in April and May 2020. We split each sod into four subplots of 50 × 50 cm (*n* = 156, Fig. 2a(iii)) and placed it in a common garden at the Botanical Garden of the Marburg University, Germany, to track the effects of changes in land‐use intensity under reduced environmental heterogeneity (Figs 2b, S1). Each of the four subplots per original plot was assigned to one of the four experimental treatments: ‘00’ (mowing once per year, LUI = 0.99), ‘0M’ (mowing twice per year, LUI = 1.4), ‘F0’ (mowing once per year and fertilizing, LUI = 2.07), and ‘FM’ (mowing twice per year and fertilizing, LUI = 2.3; Fig. 2b). Intensities of treatments of common garden subplots (numbers of cuts (cuts yr^−1^) and amount of fertilizer (kg N ha^−1^ yr^−1^)) were determined based on the 90% quantiles of land‐use management data of all plots in the Biodiversity Exploratories between 2006 and 2016 (latest data which were available at the time of planning). Experimental treatments were initiated in mid‐July 2020. Fertilization (99 kg N ha^−1^ yr^−1^) and the first mowing of the respective subplots were conducted in early summer each year, followed by the second mowing in autumn. To avoid spill‐over effects of treatments, subplots were arranged in a semi‐randomized block design with gravel in between subplots and blocks (Fig. 2b, Zieschank & Junker, 2023). Changes in land‐use intensities (∆_LUI_) were calculated by subtracting the mean LUI values (2008–2019) of the origin plots from the LUI values of common garden subplots (Δ_LUI_, Fig. 2c). Consequently, positive values indicate land‐use intensification, while negative values indicate decreases in land‐use intensity.

We recorded the taxonomic composition of vascular plant communities in June 2020 (before treatments started) and July 2022 (before second mowing). All species per subplot were identified, and the cover of each species was estimated with a resolution of 1% (Fig. 2d). On the community level, we calculated Shannon diversity for all subplots in 2020 and 2022 (R‐package vegan v.2.8‐8, Oksanen *et al*., 2024). Changes in Shannon diversity (Δ_Shannon_) were determined by subtracting the Shannon diversity of 2020 from that of 2022 (Fig. 2e). Positive values indicate increases, whereas negative values indicate decreases in species diversity; small changes indicate stability in diversity. As a measure for compositional changes, we calculated temporal β‐diversity of common garden subplots between 2020 and 2022 as the abundance‐based Bray–Curtis dissimilarities (*β*_BRAY_, i.e. higher values indicate larger compositional changes; R‐package betapart v.1.6, Baselga *et al*., 2023). To retrace whether temporal changes resulted from species loss or immigration and replacement, we also calculated temporal β‐diversity as Sørensen dissimilarity (β_SOR_, based on presence–absence data; Baselga, 2010) and partitioned it into its turnover (β_SIM_ ≙ replacement of species) and nestedness component (β_SNE_ ≙ species losses; Baselga *et al*., 2023; Fig. 2e). High β_SOR_ values reflect high temporal dissimilarity, whereas higher β_SIM_ values indicate that the temporal β‐diversity results from species replacement and immigration. Conversely, higher β_SNE_ values indicate a stronger species loss without or few replacements.

Apart from vegetation analysis, we gathered community features through digital whole‐community phenotyping (He *et al*., 2023; Zieschank & Junker, 2023; Zieschank *et al*., 2025). Specifically, we scanned subplot‐specific plant communities with a customized automated 3D plant scanning system (PlantEye F500; Phenospex, Heerlen, the Netherlands) in June 2020 (before treatments started) and in August in 2022 (before second mowing) roughly matching peak growing season in both years. After processing the scans with the software HortControl (Phenospex), we obtained aggregated, multispectral information on whole‐community level, including the NDVI of each community (Fig. 2d; Zieschank & Junker, 2023). NDVI is a well‐known proxy for community's net primary productivity (NPP) and carbon sequestration potential, two core components of ecosystem functioning, as well as plant physiological health (Pettorelli, 2013; Yang *et al*., 2022; Varouchakis *et al*., 2025). Note that NDVI and other vegetation indices derived from spectral reflectance data, such as the Enhanced Vegetation Index (EVI), relate nonlinearly to Chl content and green biomass and thus saturate (Xu *et al*., 2026). Therefore, NDVI is sensitive to a loss of functionality but less sensitive to a gain in functionality when the plateau is already reached. As our study investigates whether response diversity is a mechanism to prevent the loss of functionality, this saturation effect does not affect our primary conclusions. To estimate the functional stability of communities, we calculated subplot‐specific, absolute changes in the NDVI |∆_NDVI_| between baseline 2020 (before first treatment) and mid‐August 2022. Values close to zero suggest smaller changes in functionality (i.e., more stable), whereas higher values indicate less stability in functionality (Fig. 2e).

### Response dissimilarity *rd*_*ij*_

For the calculation of response dissimilarity *rd*_*ij*_, we used abundance–environment curves of plant species growing in communities along a land‐use intensity gradient in *n* = 150 plots within the Biodiversity Exploratories as species‐specific performance proxies (Fig. 2f(i)). Therefore, we averaged the land‐use intensity index LUI on plot‐level serving as the explanatory variable of the abundance–environment curves (2008–2022) and we calculated the mean abundances per species on plot level as dependent variable across the years 2008–2022. After ordering by LUI ascendingly, we calculated the rolling mean abundances for all plant species that occurred on *n* ≥ 25 plots (covering *n* = 96 out of *n* = 149 plant species found in the common garden) over *k* = 10 consecutive plots (R‐package runner, Kałędkowski, 2024). Thereby, we increased statistical power and reduced noise and bias via sampling variability by only considering species with sufficient numbers of observations along the gradient.

For each pair of species, we calculated the dissimilarities of the species‐specific abundance–environment curves, which serves as a measure for response dissimilarity *rd*_*ij*_ to LUI, using a copula‐based approach as implemented in the R‐package qad (Kasper *et al*., 2022; Griessenberger *et al*., 2022a). Therefore, we first calculated the mass distribution of the empirical checkerboard copulas for all given plant species with its rolling mean abundances as dependent variable and the mean LUI as the explanatory variable (both based on field data), resulting in *n* = 96 empirical copulas with a resolution of 12. As implemented in the R‐package qad, the resolution was defined as the floor function of the square root of the sample size (*n* = 150 plots), which is the mathematically proven most robust choice for setting the resolution (Junker *et al*., 2021). Afterward, *D1* distances between each pair of empirical copulas were calculated and appropriately normalized with the constant 2 to yield response dissimilarity *rd*_*ij*_ $\in \left[0,1\right],$ bounded between 0 (identical responses) and 1 (maximally different responses) (Griessenberger *et al*., 2022a,b; Junker *et al*., 2021; Trutschnig, 2011, for method validation, see Notes S1 and https://r‐qad.shinyapps.io/quantification_of_dependence/). These values were then compiled into a dissimilarity matrix. To test whether the choice of moving frame size *k* has a significant effect on the responses, we performed a Mantel test between distance matrices based on moving frame sizes *k* = 5 or *k* = 25. Results showed that response diversities are highly correlated (Mantel *r* = 0.79, *P* < 0.001), indicating that the shapes of the performance curves are insensitive to choices of *k* when it is set in a reasonable range.

Subsequently, we established a *‘response space’* based on the response dissimilarity matrix by using nonmetric multidimensional scaling (NMDS; R‐package vegan v.2.8‐8, Oksanen *et al*., 2024; Fig. 2f(ii)). Within this two‐dimensional response space, distances between species are proportional to the difference in their responses to land‐use intensity (response dissimilarities). We classified species responses to land use into three distinct categories: positively monotonic (species exhibiting an increase in abundances with increasing land‐use intensity), negatively monotonic (species exhibiting a decrease in abundance with increasing abundance), or nonmonotonic (species that do not consistently increase or decrease with increasing land‐use intensity, e.g. unimodal relationships between land‐use intensity and abundance). This response calculation was conducted using the index of monotonicity *m* as described in Hanusch *et al*. (2023), which quantifies the deviation of a given performance curve from ideal monotonicity and returning values in the interval [−1,1]. To determine the direction of association, we used Spearman's rank correlation to assign performance curves into three types: negative monotonic associations (*m* < 0; significant according to Spearman's rank correlation, ρ < 0), positive monotonic associations (*m* > 0; significant according to Spearman's rank correlation, ρ > 0), or nonmonotonic associations (*m* ~ 0, not significant according to Spearman's rank correlation), which appropriately reflected species responses to land‐use changes in common garden subplots (Fig. S2).

### Response diversity *RD*

We calculated the response diversity *RD* of plant communities in the common garden subplots as functional dispersion based on the subplot‐specific abundances of plant species recorded in 2020 or 2022 and the response dissimilarity matrix (Fig. 2g). Functional dispersion (R‐package *FD* v.1.0.12.1, Laliberté & Legendre, 2010) represents the multivariate dispersion weighted by species abundance, which is insensitive to species richness and not strongly influenced by outliers (Anderson, 2006; Laliberté & Legendre, 2010). Changes in *RD* (Δ_RD_) due to experimental treatments were obtained from subtracting *RD*_2020_ from *RD*_2022_. Positive values indicate increases, and negative values indicate decreases in *RD*. Further validation can be found in Notes S1.

### Structural equation model

We used a structural equation model (SEM) to assess and visualize the effects of land‐use intensity on response diversity as well as to analyze whether response diversity mediates the effects of land‐use intensity on ecosystem stability using the lavaan R‐package (Rosseel *et al*., 2025). SEMs are well‐suited to model and visualize complex hypotheses and mediating effects over multiple levels (McCune & Grace, 2002). We fitted the model using historical LUI and ∆_LUI_ as exogenous variables that directly affect the endogenous variables' functional stability, estimated as the absolute inverse changes in the NDVI (−1*|∆_NDVI_|), initial response diversity (*RD*_2020_), and stability in diversity (changes in species diversity Δ_Shannon_). We further modeled the direct effects of *RD* on functional stability and stability in species diversity. In addition, we computed the indirect effects of historical LUI on stability in species diversity and functional stability as the product of the respective standardized path coefficients. Total effects of historical LUI on species stability and functional stability were calculated as the sum of indirect and direct effects. The SEM was fitted using maximum likelihood estimation based on 153 of the 156 subplots (for three plots, NDVI measures are missing).

### Linear mixed‐effects models

To confirm the findings of our SEM, we also tested the relationships between *RD*, land use, and other variables characterizing the plant communities in our subplots by fitting linear mixed‐effects models (Fig. S3; R‐package lmertest v.3.1‐3, Kuznetsova *et al*., 2020). To account for the fact that each original plot (from the Biodiversity Exploratories) was split into four subplots, we used *plot origin* as random intercept to account for baseline differences (each model consisted of one fixed and one random factor). To explain *RD*, we chose a model that only contained the historical LUI as explaining variable. The model explaining Δ_*RD*_ contained the explanation term ∆_LUI_ only. Both models were tested with subsequent Type I ANOVA (Satterthwaite approximation) using the R‐package lmertest v.3.1‐3 (Kuznetsova *et al*., 2020). All models aiming for explaining community variables (β‐diversity measures, Δ_Shannon_, and |Δ_NDVI_|) contained ∆_LUI_ + *RD*_2020_ as fixed explanation terms. Afterward, we performed sequential Type I ANOVA (Satterthwaite approximation) using the same R‐package. All statistical analyses were performed using R: A language and environment for statistical computing (R Core Team, 2023).

## Results

Based on the directions of the abundance–environment relationships of *n* = 96 plant species, 47 species were classified as winners (positive relationships), 39 as losers (negative relationships), and 10 species exhibited nonmonotonic responses to land use (as indicated by the index of monotonicity *m*). Accordingly, winners, losers, and nonmonotonically responding species clustered in the response space based on response dissimilarities between species pairs (Fig. 3a). The most positively responding species was *Lolium perenne* (Fig. 3b; *m* = 0.81), whereas, for example, *Chenopodium album* showed a clear, nonmonotonic response (Fig. 3c; *m* < 0.01), and *Briza media* decreased strongest with increasing land use (Fig. 3d; *m* = −0.74).

**Fig. 3:**
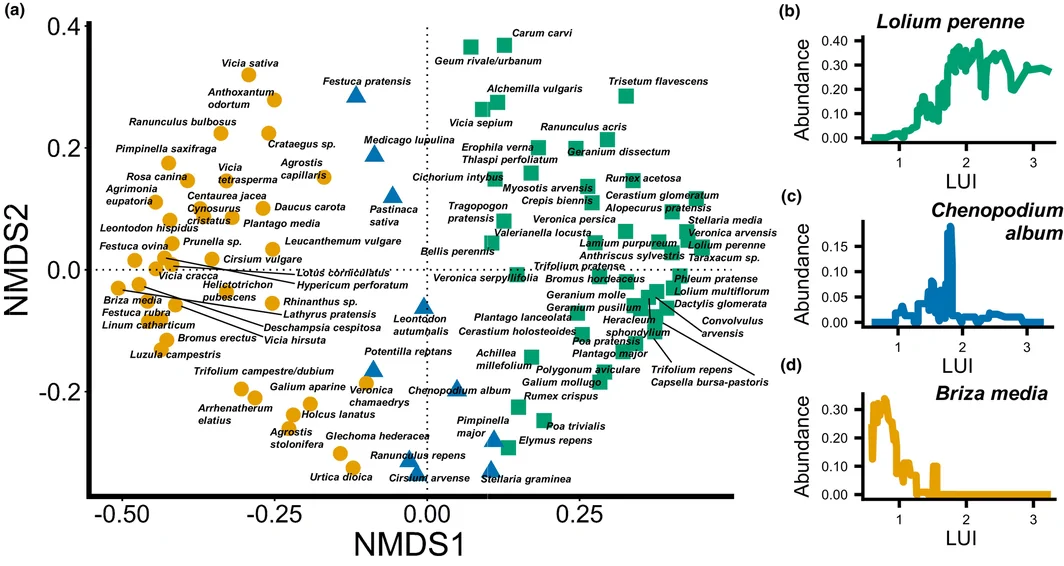
Response space of 96 grassland plant species to land‐use intensity. (a) Nonmetric multidimensional scaling (NMDS) based on response dissimilarities of species pairs separates winners (green squares, positive indices of monotonicity *m*) from losers (yellow circles, negative indices of monotonicity *m*) of land‐use intensification as well as those species that show nonmonotonic responses to land‐use intensity (blue triangles, indices of monotonicity *m* ranging around zero). (b–d) Abundance–environment relationships of three plant species: *Lolium perenne* (b, species with the most positive response to land‐use intensification), *Chenopodium album* (c, species with a clear, nonmonotonic response to land‐use intensification), and *Briza media* (d, species with the most negative response to land‐use intensification).

We used a SEM to test and visualize the relationships between land‐use intensity, response diversity, and ecosystem stability. The SEM had a good model fit (Fig. 4: *P*_chi‐square_ = 0.590; Table S1). Overall, we found clear dependence structures in our SEM (see Table S2 for regression estimates) and all significant paths could also be found in linear mixed‐effects models correcting for plot origin (Fig. S3; Table S3). Before the experimental treatments started, response diversities *RD* of the initial plant communities in the common garden subplots showed pronounced variations (mean ± SD: 0.28 ± 0.08; range: 0.01–0.42, no significant differences between treatments: ANOVA: *F*_3,152_ = 0.195, *P* = 0.900). In the SEM, *RD* was decreased by the historical land‐use intensity index LUI (the land‐use intensity communities experienced in the field before transplantation; Fig. 4; Tables S2, S3). In addition, initial Shannon diversity was also negatively associated with historical LUI, albeit not significantly (Table S3). Historical LUI and changes in land‐use intensity (Δ_LUI_) decreased the stability in species diversity in the SEM (Δ_Shannon_; Fig. 4; Table S2). However, both exogenous variables did not significantly explain functional stability (inverse |∆_NDVI_|; Fig. 4; Table S2). *RD* affected both measures of stability directly, although with different signs. Whereas *RD* positively influenced functional stability, it directly decreased stability in species diversity (Fig. 4; Table S2). Computing the indirect effects of historical LUI on functional stability revealed a weak but significant negative effect of land‐use intensity via *RD* (*z* = −2.599, *P* < 0.001; Table S2). In addition, *RD* also mediated the effects of land‐use intensity on the stability in species diversity (*z* = 3.625, *P* < 0.001). Our findings also become apparent in the linear mixed‐effects models (Fig. S3; Table S3). At the end of our experiment, the changes in response diversity Δ_RD_ were also linked to Δ_LUI_ matching the negative relationship between initial *RD* and historical LUI, that is when grass sods were exposed to increased LUI compared with their origin (positive Δ_LUI_), their *RD* decreased over the 2 yr in the common garden. The opposite was true when land‐use intensity was decreased in the common garden subplots (Fig. S3d; Table S3).

**Fig. 4:**
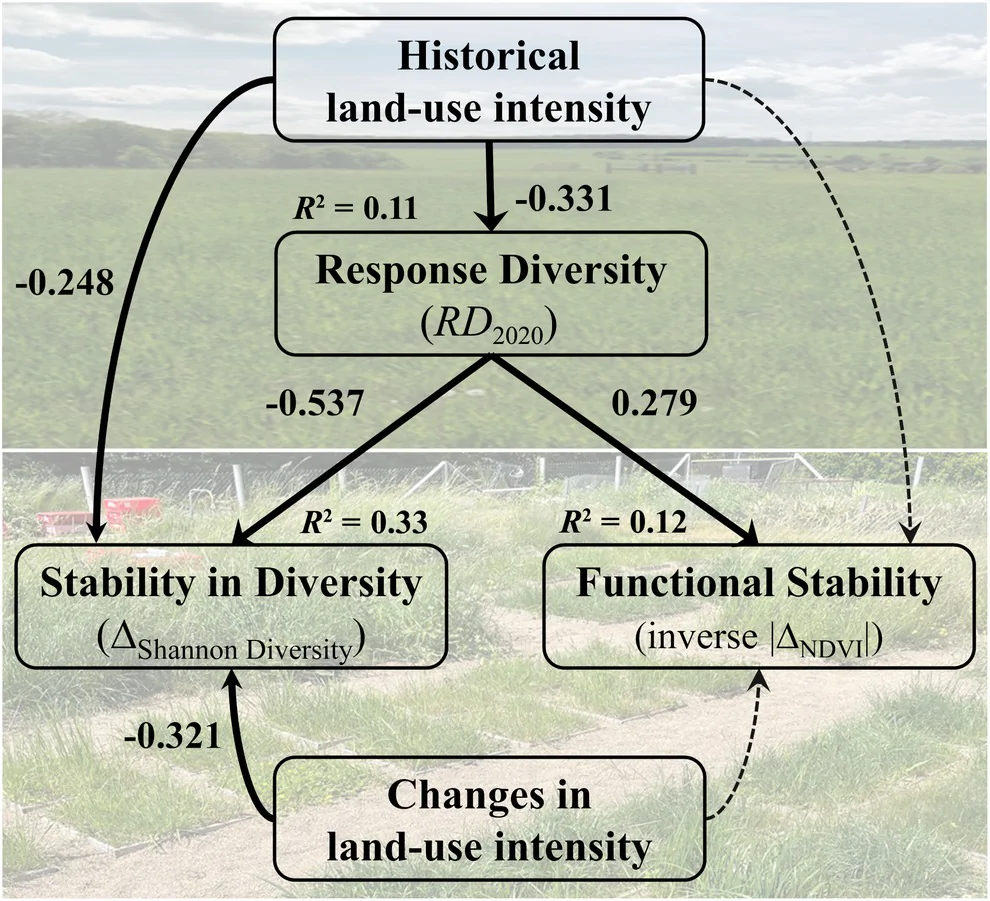
Structural equation model (SEM) visualizing the relationships between land use, response diversity, and ecosystem stability. Numbers next to arrows represent standardized path estimates and are given for significant paths only (bold arrows). Nonsignificant paths are shown as dashed arrows. Covariances are not shown. Indirect (mediated) effects of historical land‐use intensity via response diversity on functional stability (*z* = −2.599, *P* < 0.001) and stability in species diversity (*z* = 3.625, *P* < 0.001) are not shown. Statistical results are also reported in Table S2. Note that functional stability is estimated as the inverse absolute changes in Normalized Difference Vegetation Index −1*|∆_NDVI_|.

On the compositional level, abundance‐based temporal β‐diversity β_BRAY_ was positively but not significantly associated with the initial *RD* (i.e. higher *RD* led to larger changes in species composition) (Table S3) indicating that a high response diversity rather destabilizes species composition. In this context, species loss (indicated by the nestedness component of Sørensen dissimilarity) increased when initial *RD* of plant communities was higher (Fig. 5a; Table S3), whereas the turnover component (representing species replacement and immigration) was negatively related to initial *RD* (Fig. 5b; Table S3). Overall, Sørensen dissimilarity was only decreased by land‐use intensity (Table S3).

**Fig. 5:**
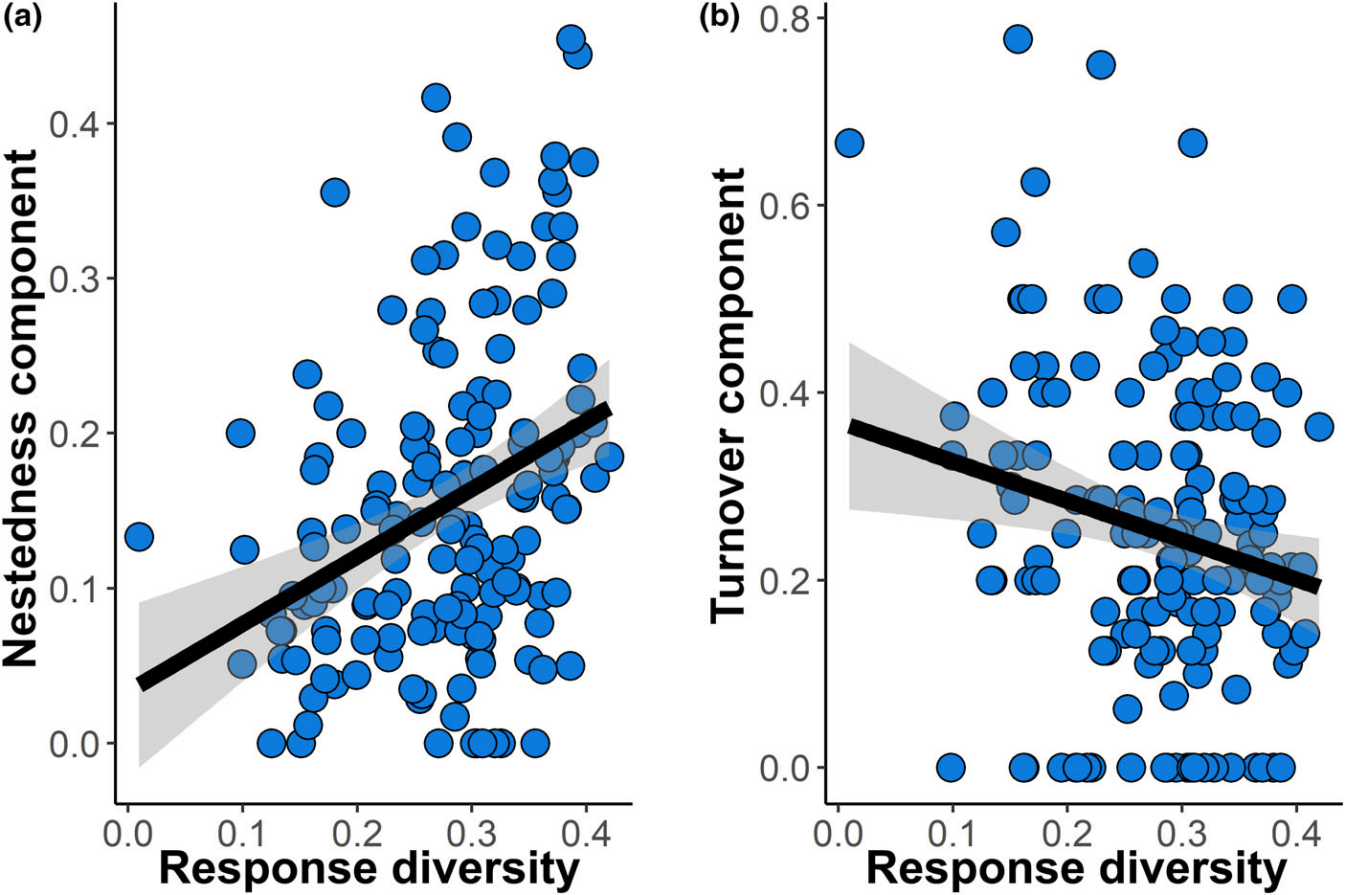
Temporal β‐diversity of community composition in response to experimental land‐use changes as a function of initial response diversity *RD*. Temporal β‐diversity of each subplot was partitioned into a turnover (= high values indicate pronounced replacement of species) and nestedness component (= high values indicate high species losses with fewer or without replacement). β‐diversity was calculated as the Sørensen index considering the presence/absence of species. (a) The nestedness component increased with increasing initial response diversity *RD* (linear mixed‐effects model; *F*_1,80.244_ = 8.62, *P* = 0.004, slope = 0.409 (95% confidence intervals: 0.182–0.636)), whereas (b) the turnover component decreased with initial response diversity *RD* (linear mixed‐effects model; *F*_1,80.591_ = 9.21, *P* = 0.003, slope = −0.511 (95% confidence intervals: −0.901 to −0.124)).

## Discussion

Our common garden experiment reveals both causes and consequences of response diversity among grassland species in the context of anthropogenic land‐use changes. Higher response diversities increased functional stability but destabilized species composition and diversity. Compositional instability in response‐diverse communities was evidenced by higher species losses without replacement compared to less response‐diverse communities. Accordingly, our hypotheses were largely supported: (H1) Overall, responses of common grassland species (estimated by abundance–environment relationships) varied substantially, ranging from negative over nonmonotonic to positive responses to land‐use intensification, which translated into species‐specific positions in the response space. Subplot‐specific compositions of species featuring either similar or dissimilar responses resulted in low or high response diversity depending on the experienced historical land‐use intensities, respectively. (H2) Land‐use intensity reduced response diversity as indicated by our field and common garden results. (H3) We found that plant communities with high response diversities were less stable in terms of species diversity and composition, but more stable in terms of functionality than plant communities with a low response diversity. (H4) Land‐use changes altered species compositions, with stronger shifts in communities that had a high response diversity before experimental treatments started. Those shifts were mainly driven by species loss which resulted in a dominant nestedness component of temporal β‐diversity and indicate that high response diversities may destabilize plant communities in terms of species composition. By contrast, communities with an initially low response diversity also tended to lose species, but those were then replaced by immigrating species, which became apparent in a strong turnover component of temporal β‐diversity.

Yachi & Loreau (1999) identified the asynchronicity of species' responses to environmental fluctuations as determinants for the insurance effects of species‐rich communities. In their model, species‐rich communities featured less variable and on average higher functionality than species‐poor communities in fluctuating environments. In times of global change, environmental conditions often do not only fluctuate but also shift constantly in one direction (Steffen *et al*., 2015), which has consequences not only for quantitative measures of species' functions but also for their persistence at local and regional scales (Hautier *et al*., 2015). Whereas extinction and immigration events are not included in Yachi and Loreau's model (Yachi & Loreau, 1999), such events are realistic and important drivers that should also influence response diversity under natural conditions (Elmqvist *et al*., 2003). Recently, the importance of a stably coexisting species (so‐called core species) for ecosystem functioning has been highlighted (Allan *et al*., 2025). Despite conceptual differences (i.e. the number of core species is related to increased functionality via complementary effects (Godoy *et al*., 2020) and response diversity addresses compensatory effects), the sets of stably coexisting species have been linked to differing responses to environmental conditions before (Magurran & Henderson, 2003). In this context, environmental changes such as increased or decreased land‐use intensity may support different sets of stably coexisting species (depending on their responses to the change) ultimately ensuring and stabilizing productivity (Fournier *et al*., 2017; Snell Taylor *et al*., 2018). For instance, land‐use intensification leads not only to changes in productivity (Allan *et al*., 2015; Schils *et al*., 2022) but also to shifts in species composition and decreased diversity (Weiner *et al*., 2011; Gossner *et al*., 2016), which might be even more challenging for ecosystems when resistance and recovery are further reduced by constant pressures in comparison with fluctuating conditions. Aligning with the original hypothesis (Yachi & Loreau, 1999), reductions in land use also caused increases in response diversity over two growing seasons indicating that ecosystems have the potential to recover under decreased stresses not only in diversity and functionality but also in their capacity to functionally tolerate future stresses.

While theoretical and conceptual work on the insurance hypothesis and response diversity usually addresses functional stability (Yachi & Loreau, 1999; Mori *et al*., 2013), empirical studies considered additional measures for stability reflecting its multidimensionality (Donohue *et al*., 2016). These measures for stability included the variance of functional diversity based on response traits (Laliberté *et al*., 2010; Aquilué *et al*., 2020), the persistence of species and thus species composition and diversity (Ives *et al*., 1999; Chillo *et al*., 2011; Baskett *et al*., 2014), quantitative measures of ecosystem functions (Cariveau *et al*., 2013; Moore & Olden, 2017; Sasaki *et al*., 2019), and proxies for ecosystem functions (Mazzochini *et al*., 2024).

In this context, our results suggest a stabilizing effect of response diversity on ecosystem functions under global change, directly validating Yachi & Loreau's theory (Yachi & Loreau, 1999). By contrast, our findings also indicate that species composition and diversity are dimensions of stability that decreased under high response diversity suggesting that functional compensation may come along with species losses in response‐diverse communities (i.e. winning species replace functionally similar loser species). Latest findings within the Biodiversity Exploratories have also identified functional trait diversity and associated resource‐use strategies as further drivers of community stability (Neyret *et al*., 2024; Sperandii *et al*., 2025) showing that multiple mechanisms of diversity can contribute to the relationship between diversity and ecosystem functioning. Sperandii *et al*. (2025) highlighted the importance of plants' functional strategies – known as the plants' spectrum of form and function (Díaz *et al*., 2016) – for community stability, whereas functional diversity just played a minor but positive role in grasslands. Thereby, land‐use intensity influenced the acquisitive‐conservative trait spectrum with negative consequences for community stability via decreased species richness and increased population variability. In this context, Busch *et al*. also linked plant species benefiting from land‐use intensification to more acquisitive trait strategies in the Exploratories before (2019).

Our data confirm previous results separating plant species into those that respond negatively, positively (Busch *et al*., 2019), or do not show a monotonic response to an environmental gradient (Hanusch *et al*., 2023), albeit with differing proportions. Whereas the presence of winner and loser species in a community predetermines community responses to land‐use changes that favor the one or the other group of plants (Socher *et al*., 2013; Gilhaus *et al*., 2017; Kachler *et al*., 2023), nonmonotonic responses have been reported to stabilize the diversity and functionality of ecosystems (Zhang *et al*., 2015; Yan & Zhang, 2018). In this context, response spaces as introduced in this study do not just help to classify groups of species with similar responses but also to identify species that are particularly threatened by global change components. Future studies should validate our findings under more natural conditions with multiple global change components acting simultaneously on vegetation stability, which may alter effects on grasslands (Pompe *et al*., 2008; de Chazal & Rounsevell, 2009; Bütof *et al*., 2012).

Our results, along with variable reported net effects of response diversity on ecosystem stability ranging from positive (Sasaki *et al*., 2019; Aquilué *et al*., 2020) to no effects (Cariveau *et al*., 2013; Elsalahy *et al*., 2020), indicate that the stabilizing effect of response diversity may be system‐specific and depends on how stability is defined. Furthermore, and maybe most importantly, the nature of environmental change (i.e. temporal fluctuating conditions and unidirectional increases in environmental parameters) may strongly affect the relationship between response diversity and ecosystem stability (Donohue *et al*., 2016; Kéfi *et al*., 2019). Additionally, as our data demonstrate, the magnitude of change clearly affects stability and the buffering effect of response diversity (Donohue *et al*., 2016). Finally, most studies, including ours, focus on a single changing environmental factor or stress (Donohue *et al*., 2016; Kéfi *et al*., 2019; Ross *et al*., 2023). Most ecosystems, however, are challenged by changes in multiple factors or multifactorial stress combinations that may act synchronously or sequentially (Zandalinas & Mittler, 2022). It is questionable whether previously stressed communities with consequently reduced response diversities are still sufficiently resistant or resilient to cope with future stresses. In this context, future studies may specifically test whether species that are tolerant to one stressor are also tolerant to another stressor, or whether species feature tolerance to a specific set of stressors while they cannot tolerate others. If species can be divided into those with a universal tolerance and those with rather low thresholds of stress tolerance, communities may quickly loose considerable components of their biodiversity.

We conclude that response diversity is an important determinant for ecosystem stability that mechanistically links functional and compositional stability. Performance–environment relationships turned out to be a powerful tool for assessing pairwise response dissimilarities and response diversity that do not depend on proxies for species responses to environmental changes (see Ross *et al*., 2023; Ross & Sasaki, 2024). However, the strong context dependency of response diversity–stability relationships requires system‐specific definitions of responses as well as of stability in order to adequately measure and take full advantage of this dimension of diversity. Further insights into the role of multifactorial stress combinations as well as the nature and magnitude of change in affecting response diversity and ecosystem stability are clearly required to develop a robust conceptual framework, and to design conservation strategies that facilitate resilience and resistance and thus maintain ecosystem functioning.

## Competing interests

None declared.

## Author contributions

RRJ conceived the study. MP, VZ and RRJ designed the study. VZ performed fieldwork. MP, VZ, WT and RRJ performed statistical analysis. MP, VZ and RRJ wrote the manuscript. All authors contributed to the final version of the manuscript.

## Disclaimer

The New Phytologist Foundation remains neutral with regard to jurisdictional claims in maps and in any institutional affiliations.

## Supporting information


**Fig. S1** Detailed experimental setup of the common garden.
**Fig. S2** Comparison of species responses to land‐use intensification in field and common garden.
**Fig. S3** Community responses to land‐use changes in the field and common garden plots as a function of response diversity *RD*_2020_.
**Methods S1** Equation for the calculation of the standardized land‐use intensity index LUI (historical LUI) which was further used to calculate ∆LUI.
**Methods S2** Equation for the calculation of the standardized land‐use intensity index LUI used to create abundance–environment relationships (2008–2022).
**Notes S1** Detailed description of the R‐package qad and a simulation which verifies the suitability of qad to quantify response dissimilarities and response diversity.
**Table S1** Structural equation model fit parameters.
**Table S2** Regression estimates for structural equation model.
**Table S3** ANOVA results of all tested linear mixed‐effects models.
Please note: Wiley is not responsible for the content or functionality of any Supporting Information supplied by the authors. Any queries (other than missing material) should be directed to the *New Phytologist* Central Office.

## Data Availability

This work was performed within the scope of the Biodiversity Exploratories program (DFG Priority Program 1374). Long‐term field data were recorded by the Botany Core project of the Biodiversity Exploratories program (DFG Priority Program 1374) and are publicly available in the Biodiversity Exploratories Information System (doi: 10.17616/R32P9Q). The dataset is listed in the references section (Hinderling & Keller, 2023). The dataset containing long‐term field data collected by the Botany Core project of the Biodiversity Exploratories can be found under the following link: https://www.bexis.uni‐jena.de/ddm/data/Showdata/31389. The datasets created within the project ‘EXClAvE’ containing data about common garden subplots can be found at https://www.bexis.uni‐jena.de/ddm/data/Showdata/31896 and https://www.bexis.uni‐jena.de/ddm/data/Showdata/31269. The R‐codes used for statistical analyses and method validation via simulation as well as five data sets can be found at figshare: doi: 10.6084/m9.figshare.32187945.

## References

[nph71546-bib-0001] Allan E , Manning P , Alt F , Binkenstein J , Blaser S , Blüthgen N , Böhm S , Grassein F , Hölzel N , Klaus VH *et al*. 2015. Land use intensification alters ecosystem multifunctionality via loss of biodiversity and changes to functional composition. Ecology Letters 18: 834–843. 10.1111/ele.12469 PMC4744976 26096863

[nph71546-bib-0002] Allan E , Penone C , Schmid B , Godoy O , Pichon NA . 2025. When can we expect negative effects of plant diversity on community biomass? Journal of Ecology 113: 1955–1969.

[nph71546-bib-0003] Anderson MJ . 2006. Distance‐based tests for homogeneity of multivariate dispersions. Biometrics 62: 245–253. 10.1111/j.1541-0420.2005.00440.x 16542252

[nph71546-bib-0004] Aquilué N , Filotas É , Craven D , Fortin M , Brotons L , Messier C . 2020. Evaluating forest resilience to global threats using functional response traits and network properties. Ecological Applications 30: e02095. 10.1002/eap.2095 32080941

[nph71546-bib-0005] Baselga A . 2010. Partitioning the turnover and nestedness components of beta diversity. Global Ecology and Biogeography 19: 134–143.

[nph71546-bib-0006] Baselga A , Orme D , Villeger S , De Bortoli J , Leprieur F , Logez M , Martinez‐Santalla S , Martin‐Devasa R , Gomez‐Rodriguez C , Crujeiras RM . 2023. betapart: partitioning beta diversity into turnover and nestedness components 1.6. [WWW document] URL http://CRAN.R‐project.org/package=betapart [accessed 21 October 2024].

[nph71546-bib-0007] Baskett ML , Fabina NS , Gross K . 2014. Response diversity can increase ecological resilience to disturbance in coral reefs. The American Naturalist 184: E16–E31. 10.1086/676643 25058289

[nph71546-bib-0008] Bazzichetto M , Sperandii MG , Penone C , Keil P , Allan E , Lepš J , Prati D , Fischer M , Bolliger R , Gossner MM *et al*. 2024. Biodiversity promotes resistance but dominant species shape recovery of grasslands under extreme drought. Journal of Ecology 112: 1087–1100.

[nph71546-bib-0009] Bhaskar R , Arreola F , Mora F , Martinez‐Yrizar A , Martinez‐Ramos M , Balvanera P . 2018. Response diversity and resilience to extreme events in tropical dry secondary forests. Forest Ecology and Management 426: 61–71.

[nph71546-bib-0010] Blüthgen N , Dormann CF , Prati D , Klaus VH , Kleinebecker T , Hölzel N , Alt F , Boch S , Gockel S , Hemp A *et al*. 2012. A quantitative index of land‐use intensity in grasslands: Integrating mowing, grazing and fertilization. Basic and Applied Ecology 13: 207–220.

[nph71546-bib-0011] Blüthgen N , Simons NK , Jung K , Prati D , Renner SC , Boch S , Fischer M , Hölzel N , Klaus VH , Kleinebecker T *et al*. 2016. Land use imperils plant and animal community stability through changes in asynchrony rather than diversity. Nature Communications 7: 10697. 10.1038/ncomms10697 PMC4754335 26869180

[nph71546-bib-0012] Boulangeat I , Lavergne S , Van Es J , Garraud L , Thuiller W . 2012. Niche breadth, rarity and ecological characteristics within a regional flora spanning large environmental gradients. Journal of Biogeography 39: 204–214.

[nph71546-bib-0013] Busch V , Klaus VH , Schäfer D , Prati D , Boch S , Müller J , Chisté M , Mody K , Blüthgen N , Fischer M *et al*. 2019. Will I stay or will I go? Plant species‐specific response and tolerance to high land‐use intensity in temperate grassland ecosystems. Journal of Vegetation Science 30: 674–686.

[nph71546-bib-0014] Bütof A , von Riedmatten LR , Dormann CF , Scherer‐Lorenzen M , Welk E , Bruelheide H . 2012. The responses of grassland plants to experimentally simulated climate change depend on land use and region. Global Change Biology 18: 127–137.

[nph71546-bib-0015] Cadotte MW . 2017. Functional traits explain ecosystem function through opposing mechanisms. Ecology Letters 20: 989–996. 10.1111/ele.12796 28639274

[nph71546-bib-0016] Cardinale BJ , Srivastava DS , Emmett Duffy J , Wright JP , Downing AL , Sankaran M , Jouseau C . 2006. Effects of biodiversity on the functioning of trophic groups and ecosystems. Nature 443: 989–992. 10.1038/nature05202 17066035

[nph71546-bib-0017] Cariveau DP , Williams NM , Benjamin FE , Winfree R . 2013. Response diversity to land use occurs but does not consistently stabilise ecosystem services provided by native pollinators. Ecology Letters 16: 903–911. 10.1111/ele.12126 23692675

[nph71546-bib-0018] de Chazal J , Rounsevell MDA . 2009. Land‐use and climate change within assessments of biodiversity change: a review. Global Environmental Change 19: 306–315.

[nph71546-bib-0019] Chillo V , Anand M , Ojeda RA . 2011. Assessing the use of functional diversity as a measure of ecological resilience in arid rangelands. Ecosystems 14: 1168–1177.

[nph71546-bib-0020] Craven D , Filotas E , Angers VA , Messier C . 2016. Evaluating resilience of tree communities in fragmented landscapes: linking functional response diversity with landscape connectivity. Diversity and Distributions 22: 505–518.

[nph71546-bib-0021] Díaz S , Cabido M . 2001. Vive la différence: plant functional diversity matters to ecosystem processes. Trends in Ecology & Evolution 16: 646–655.

[nph71546-bib-0022] Díaz S , Kattge J , Cornelissen JHC , Wright IJ , Lavorel S , Dray S , Reu B , Kleyer M , Wirth C , Colin Prentice I *et al*. 2016. The global spectrum of plant form and function. Nature 529: 167–171. 10.1038/nature16489 26700811

[nph71546-bib-0023] Díaz S , Settele J , Brondízio ES , Ngo HT , Guèze M , Agard J , Almuth A , Balvanera P , Brauman K , Butchart S *et al*. 2019. Summary for policymakers of the global assessment report on biodiversity and ecosystem services of the Intergovernmental Science‐Policy Platform on Biodiversity and Ecosystem Services. Bonn, Germany: IPBES Secretariat, 56.

[nph71546-bib-0024] Donohue I , Hillebrand H , Montoya JM , Petchey OL , Pimm SL , Fowler MS , Healy K , Jackson AL , Lurgi M , McClean D *et al*. 2016. Navigating the complexity of ecological stability. Ecology Letters 19: 1172–1185. 10.1111/ele.12648 27432641

[nph71546-bib-0025] Donohue I , Petchey OL , Montoya JM , Jackson AL , McNally L , Viana M , Healy K , Lurgi M , O'Connor NE , Emmerson MC . 2013. On the dimensionality of ecological stability. Ecology Letters 16: 421–429. 10.1111/ele.12086 23419041

[nph71546-bib-0026] Elmqvist T , Folke C , Nyström M , Peterson G , Bengtsson J , Walker B , Norberg J . 2003. Response diversity, ecosystem change, and resilience. Frontiers in Ecology and the Environment 1: 488–494.

[nph71546-bib-0027] Elsalahy HH , Bellingrath‐Kimura SD , Roß C‐L , Kautz T , Döring TF . 2020. Crop resilience to drought with and without response diversity. Frontiers in Plant Science 11: 123. 10.3389/fpls.2020.00721 PMC7283915 32582251

[nph71546-bib-0028] Fischer M , Bossdorf O , Gockel S , Hänsel F , Hemp A , Hessenmöller D , Korte G , Nieschulze J , Pfeiffer S , Prati D *et al*. 2010. Implementing large‐scale and long‐term functional biodiversity research: the biodiversity exploratories. Basic and Applied Ecology 11: 473–485.

[nph71546-bib-0029] Foley JA , DeFries R , Asner GP , Barford C , Bonan G , Carpenter SR , Chapin FS , Coe MT , Daily GC , Gibbs HK *et al*. 2005. Global consequences of land use. Science 309: 570–574. 10.1126/science.1111772 16040698

[nph71546-bib-0030] Fournier B , Mouquet N , Leibold MA , Gravel D . 2017. An integrative framework of coexistence mechanisms in competitive metacommunities. Ecography 40: 630–641.

[nph71546-bib-0031] Gilhaus K , Boch S , Fischer M , Hölzel N , Kleinebecker T , Prati D , Rupprecht D , Schmitt B , Klaus VH . 2017. Grassland management in Germany: effects on plant diversity and vegetation composition. Tuexenia 37: 379–397.

[nph71546-bib-0032] Godoy O , Gómez‐Aparicio L , Matías L , Pérez‐Ramos IM , Allan E . 2020. An excess of niche differences maximizes ecosystem functioning. Nature Communications 11: 4180. 10.1038/s41467-020-17960-5 PMC7442808 32826915

[nph71546-bib-0033] Gossner MM , Lewinsohn TM , Kahl T , Grassein F , Boch S , Prati D , Birkhofer K , Renner SC , Sikorski J , Wubet T *et al*. 2016. Land‐use intensification causes multitrophic homogenization of grassland communities. Nature 540: 266–269. 10.1038/nature20575 27919075

[nph71546-bib-0034] Griessenberger F , Junker RR , Trutschnig W . 2022a. On a multivariate copula‐based dependence measure and its estimation. Electronic Journal of Statistics 16: 2206–2251.

[nph71546-bib-0035] Griessenberger F , Trutschnig W , Junker RR . 2022b. qad: An R‐package to detect asymmetric and directed dependence in bivariate samples. Methods in Ecology and Evolution 13: 2138–2149.

[nph71546-bib-0036] Hanusch M , He X , Janssen S , Selke J , Trutschnig W , Junker RR . 2023. Exploring the frequency and distribution of ecological non‐monotonicity in associations among ecosystem constituents. Ecosystems 26: 1819–1840. 10.1007/s10021-023-00867-9 PMC10721710 38106357

[nph71546-bib-0037] Hautier Y , Tilman D , Isbell F , Seabloom EW , Borer ET , Reich PB . 2015. Anthropogenic environmental changes affect ecosystem stability via biodiversity. Science 348: 336–340. 10.1126/science.aaa1788 25883357

[nph71546-bib-0038] He X , Hanusch M , Saueressig L , Schriever A , Seifert T , Villhauer H , Zieschank V , Junker RR . 2023. Tracking succession by means of 3D scans of plant communities in a glacier forefield to infer assembly processes. Oikos 2023: e10095.

[nph71546-bib-0039] Hinderling J , Keller S . 2023. Vegetation records for grassland EPs, 2008 ‐ 2022. Version 1. Biodiversity Exploratories Information System. Dataset. [WWW document] URL www.bexis.uni‐jena.de/ddm/data/Showdata/31389?tag=1.%20Dataset%20ID=%2031389 [accessed 11 March 2025].

[nph71546-bib-0040] Hooper DU , Chapin FS , Ewel JJ , Hector A , Inchausti P , Lavorel S , Lawton JH , Lodge DM , Loreau M , Naeem S *et al*. 2005. Effects of biodiversity on ecosystem functioning: a consensus of current knowledge. Ecological Monographs 75: 3–35.

[nph71546-bib-0041] Ives AR , Gross K , Klug JL . 1999. Stability and variability in competitive communities. Science 286: 542–544. 10.1126/science.286.5439.542 10521351

[nph71546-bib-0042] Junker RR , Griessenberger F , Trutschnig W . 2021. Estimating scale‐invariant directed dependence of bivariate distributions. Computational Statistics & Data Analysis 153: 107058.

[nph71546-bib-0043] Kachler J , Benra F , Bolliger R , Isaac R , Bonn A , Felipe‐Lucia MR . 2023. Can we have it all? The role of grassland conservation in supporting forage production and plant diversity. Landscape Ecology 38: 4451–4465.

[nph71546-bib-0044] Kałędkowski D . 2024. runner: running operations for vectors. R package v.0.4.4. [WWW document] URL https://cran.r‐project.org/package=runner [accessed 21 October 2024].

[nph71546-bib-0045] Kasper T , Griessenberger F , Junker RR , Petzel V , Trutschnig W . 2022. qad: quantification of asymmetric dependence. R package v.1.0.4. [WWW document] URL https://cran.rproject.org/web/packages/qad/index.html [accessed 5 February 2025].

[nph71546-bib-0046] Kéfi S , Domínguez‐García V , Donohue I , Fontaine C , Thébault E , Dakos V . 2019. Advancing our understanding of ecological stability. Ecology Letters 22: 1349–1356. 10.1111/ele.13340 31286641

[nph71546-bib-0047] Kuznetsova A , Brockhoff PB , Christensen RHB . 2020. lmertest package: tests in linear mixed effects models. v.3.1‐3. [WWW document] URL https://cran.r‐project.org/web/packages/lmerTest/index.html [accessed 7 May 2025].

[nph71546-bib-0048] Laliberté E , Legendre P . 2010. A distance‐based framework for measuring functional diversity from multiple traits. Ecology 91: 299–305. 10.1890/08-2244.1 20380219

[nph71546-bib-0049] Laliberté E , Wells JA , DeClerck F , Metcalfe DJ , Catterall CP , Queiroz C , Aubin I , Bonser SP , Ding Y , Fraterrigo JM *et al*. 2010. Land‐use intensification reduces functional redundancy and response diversity in plant communities. Ecology Letters 13: 76–86. 10.1111/j.1461-0248.2009.01403.x 19917052

[nph71546-bib-0050] Laughlin DC . 2014. Applying trait‐based models to achieve functional targets for theory‐driven ecological restoration. Ecology Letters 17: 771–784. 10.1111/ele.12288 24766299

[nph71546-bib-0051] Leary DJ , Petchey OL . 2009. Testing a biological mechanism of the insurance hypothesis in experimental aquatic communities. Journal of Animal Ecology 78: 1143–1151. 10.1111/j.1365-2656.2009.01586.x 19594662

[nph71546-bib-0052] Lisner A , Konečná M , Blažek P , Lepš J . 2023. Community biomass is driven by dominants and their characteristics – the insight from a field biodiversity experiment with realistic species loss scenario. Journal of Ecology 111: 240–250.

[nph71546-bib-0053] Loreau M , De Mazancourt C . 2013. Biodiversity and ecosystem stability: a synthesis of underlying mechanisms. Ecology Letters 16: 106–115. 10.1111/ele.12073 23346947

[nph71546-bib-0054] Magurran AE , Henderson PA . 2003. Explaining the excess of rare species in natural species abundance distributions. Nature 422: 714–716. 10.1038/nature01547 12700760

[nph71546-bib-0055] Mazzochini GG , Rowland L , Lira‐Martins D , FV B , Flores BM , Hirota M , Pennington RT , Oliveira RS . 2024. Spectral asynchrony as a measure of ecosystem response diversity. Global Change Biology 30: e17174.

[nph71546-bib-0056] McCann KS . 2000. The diversity–stability debate. Nature 405: 228–233. 10.1038/35012234 10821283

[nph71546-bib-0057] McCann MJ . 2016. Response diversity of free‐floating plants to nutrient stoichiometry and temperature: growth and resting body formation. PeerJ 4: e1781. 10.7717/peerj.1781 PMC4793347 26989619

[nph71546-bib-0058] McCune B , Grace J . 2002. Structural equation modeling. In: Analysis of ecological communities. Gleneden Beach, OR, USA: MjM Software Design, 233–256.

[nph71546-bib-0059] Moore JW , Olden JD . 2017. Response diversity, nonnative species, and disassembly rules buffer freshwater ecosystem processes from anthropogenic change. Global Change Biology 23: 1871–1880. 10.1111/gcb.13536 27761971

[nph71546-bib-0060] Mori AS , Furukawa T , Sasaki T . 2013. Response diversity determines the resilience of ecosystems to environmental change. Biological Reviews 88: 349–364. 10.1111/brv.12004 23217173

[nph71546-bib-0061] Nash KL , Graham NAJ , Jennings S , Wilson SK , Bellwood DR . 2016. Herbivore cross‐scale redundancy supports response diversity and promotes coral reef resilience. Journal of Applied Ecology 53: 646–655.

[nph71546-bib-0062] Newbold T , Hudson LN , Hill SLL , Contu S , Lysenko I , Senior RA , Börger L , Bennett DJ , Choimes A , Collen B *et al*. 2015. Global effects of land use on local terrestrial biodiversity. Nature 520: 45–50. 10.1038/nature14324 25832402

[nph71546-bib-0063] Neyret M , Le Provost G , Boesing AL , Schneider FD , Baulechner D , Bergmann J , de Vries FT , Fiore‐Donno AM , Geisen S , Goldmann K *et al*. 2024. A slow‐fast trait continuum at the whole community level in relation to land‐use intensification. Nature Communications 15: 1251. 10.1038/s41467-024-45113-5 PMC10858939 38341437

[nph71546-bib-0064] Nonaka S , Sasaki T . 2025. Species asynchrony, species stability, and compositional stability jointly stabilize, while bryophyte cover destabilizes plant communities across Japanese moorlands. Science of the Total Environment 969: 178955. 10.1016/j.scitotenv.2025.178955 40020582

[nph71546-bib-0065] Oksanen J , Simpson GL , Blanchet FG , Kindt R , Legendre P , Minchin PR , O'Hara RB , Solymos P , Stevens MHH , Szoecs E *et al*. 2024. vegan: community ecology package. v.2.6‐8. [WWW document] URL https://cran.r‐project.org/web/packages/vegan/vegan.pdf [accessed 28 August 2024].

[nph71546-bib-0066] Ostrowski A , Lorenzen K , Petzold E , Schindler S . 2020. Land use intensity index (LUI) calculation tool of the Biodiversity Exploratories project for grassland survey data from three different regions in Germany since 2006, BEXIS 2 module. doi: 10.5281/zenodo.3865579.

[nph71546-bib-0067] Pettorelli N . 2013. The normalized difference vegetation index. Oxford, UK: OUP.

[nph71546-bib-0068] Pimm SL . 1984. The complexity and stability of ecosystems. Nature 307: 321–326.

[nph71546-bib-0069] Polazzo F , Limberger R , Pennekamp F , Ross SRP‐J , Simpson GL , Petchey OL . 2024. Measuring the response diversity of ecological communities experiencing multifarious environmental change. Global Change Biology 30: e17594. 10.1111/gcb.17594 PMC11580112 39569752

[nph71546-bib-0070] Pompe S , Hanspach J , Badeck F , Klotz S , Thuiller W , Kühn I . 2008. Climate and land use change impacts on plant distributions in Germany. Biology Letters 4: 564–567. 10.1098/rsbl.2008.0231 PMC2610074 18664416

[nph71546-bib-0071] R Core Team . 2023. R: a language and environment for statistical computing. Vienna, Austria: R Foundation for Statistical Computing.

[nph71546-bib-0072] Ross SRP‐J , Petchey OL , Sasaki T , Armitage DW . 2023. How to measure response diversity. Methods in Ecology and Evolution 14: 1150–1167.

[nph71546-bib-0073] Ross SRP‐J , Sasaki T . 2024. Limited theoretical and empirical evidence that response diversity determines the resilience of ecosystems to environmental change. Ecological Research 39: 115–130. 10.1111/brv.12004 23217173

[nph71546-bib-0074] Rosseel Y , Jorgensen TD , Wilde LD , Oberski D , Byrnes J , Vanbrabant L , Savalei V , Merkle E , Hallquist M , Rhemtulla M *et al*. 2025. lavaan: latent variable analysis. [WWW document] URL https://cran.r‐project.org/web/packages/lavaan/index.html [accessed 19 November 2025].

[nph71546-bib-0075] Ruiz‐Moreno A , Emslie MJ , Connolly SR . 2024. High response diversity and conspecific density‐dependence, not species interactions, drive dynamics of coral reef fish communities. Ecology Letters 27: e14424. 10.1111/ele.14424 38634183

[nph71546-bib-0076] Sasaki T , Lu X , Hirota M , Bai Y . 2019. Species asynchrony and response diversity determine multifunctional stability of natural grasslands. Journal of Ecology 107: 1862–1875.

[nph71546-bib-0077] Schils RLM , Bufe C , Rhymer CM , Francksen RM , Klaus VH , Abdalla M , Milazzo F , Lellei‐Kovács E , ten BH , Bertora C *et al*. 2022. Permanent grasslands in Europe: land use change and intensification decrease their multifunctionality. Agriculture, Ecosystems & Environment 330: 107891.

[nph71546-bib-0078] Schnabel F , Liu X , Kunz M , Barry KE , Bongers FJ , Bruelheide H , Fichtner A , Härdtle W , Li S , Pfaff C‐T *et al*. 2021. Species richness stabilizes productivity via asynchrony and drought‐tolerance diversity in a large‐scale tree biodiversity experiment. Science Advances 7: eabk1643. 10.1126/sciadv.abk1643 PMC8682986 34919425

[nph71546-bib-0079] Snell Taylor SJ , Evans BS , White EP , Hurlbert AH . 2018. The prevalence and impact of transient species in ecological communities. Ecology 99: 1825–1835. 10.1002/ecy.2398 29802772

[nph71546-bib-0080] Socher SA , Prati D , Boch S , Müller J , Baumbach H , Gockel S , Hemp A , Schöning I , Wells K , Buscot F *et al*. 2013. Interacting effects of fertilization, mowing and grazing on plant species diversity of 1500 grasslands in Germany differ between regions. Basic and Applied Ecology 14: 126–136.

[nph71546-bib-0081] Sperandii MG , Bazzichetto M , Götzenberger L , Moretti M , Achury R , Blüthgen N , Fischer M , Hölzel N , Klaus VH , Kleinebecker T *et al*. 2025. Functional traits mediate the effect of land use on drivers of community stability within and across trophic levels. Science Advances 11: eadp6445. 10.1126/sciadv.adp6445 PMC11759044 39854460

[nph71546-bib-0082] Stavert JR , Pattemore DE , Gaskett AC , Beggs JR , Bartomeus I . 2017. Exotic species enhance response diversity to land‐use change but modify functional composition. Proceedings of the Royal Society B: Biological Sciences 284: 20170788. 10.1098/rspb.2017.0788 PMC5563801 28794218

[nph71546-bib-0083] Steffen W , Broadgate W , Deutsch L , Gaffney O , Ludwig C . 2015. The trajectory of the Anthropocene: The Great Acceleration. The Anthropocene Review 2: 81–98.

[nph71546-bib-0084] Tilman D , Reich PB , Knops JMH . 2006. Biodiversity and ecosystem stability in a decade‐long grassland experiment. Nature 441: 629–632. 10.1038/nature04742 16738658

[nph71546-bib-0085] Trutschnig W . 2011. On a strong metric on the space of copulas and its induced dependence measure. Journal of Mathematical Analysis and Applications 384: 690–705.

[nph71546-bib-0086] Van Ruijven J , Berendse F . 2010. Diversity enhances community recovery, but not resistance, after drought. Journal of Ecology 98: 81–86.

[nph71546-bib-0087] Varouchakis EA , Komnitsas K , Galetakis M . 2025. Spatiotemporal analysis of vegetation health and moisture dynamics in rehabilitated mining quarries using satellite imagery. Environmental Processes 12: 45.

[nph71546-bib-0088] Vogt J , Klaus V , Both S , Fürstenau C , Gockel S , Gossner M , Heinze J , Hemp A , Hölzel N , Jung K *et al*. 2019. Eleven years' data of grassland management in Germany. Biodiversity Data Journal 7: e36387. 10.3897/BDJ.7.e36387 PMC6778154 31598068

[nph71546-bib-0089] Wagg C , Roscher C , Weigelt A , Vogel A , Ebeling A , de Luca E , Roeder A , Kleinspehn C , Temperton VM , Meyer ST *et al*. 2022. Biodiversity–stability relationships strengthen over time in a long‐term grassland experiment. Nature Communications 13: 7752. 10.1038/s41467-022-35189-2 PMC9751076 36517483

[nph71546-bib-0090] Walker B , Kinzig A , Langridge J . 1999. Plant attribute diversity, resilience, and ecosystem function: the nature and significance of dominant and minor species. Ecosystems 2: 95–113.

[nph71546-bib-0091] Wang Y , Cadotte MW , Chen Y , Fraser LH , Zhang Y , Huang F , Luo S , Shi N , Loreau M . 2019. Global evidence of positive biodiversity effects on spatial ecosystem stability in natural grasslands. Nature Communications 10: 3207. 10.1038/s41467-019-11191-z PMC6642091 31324792

[nph71546-bib-0092] Weiner CN , Werner M , Linsenmair KE , Blüthgen N . 2011. Land use intensity in grasslands: changes in biodiversity, species composition and specialisation in flower visitor networks. Basic and Applied Ecology 12: 292–299.

[nph71546-bib-0093] Wesche K , Krause B , Culmsee H , Leuschner C . 2012. Fifty years of change in Central European grassland vegetation: large losses in species richness and animal‐pollinated plants. Biological Conservation 150: 76–85.

[nph71546-bib-0094] Winfree R , Kremen C . 2009. Are ecosystem services stabilized by differences among species? A test using crop pollination. Proceedings of the Royal Society B: Biological Sciences 276: 229–237. 10.1098/rspb.2008.0709 PMC2674338 18796401

[nph71546-bib-0095] Xu D‐F , Yuan Q , Guo H‐Q , Tan B , Quan F , Yu Z‐X , Han D‐Y , Lu Y‐J , Hao Y‐Y , Zhou S‐Q *et al*. 2026. Adaptive seasonal management after eradicating invasive *Spartina*: a nature‐based approach. Ecological Engineering 231: 108071.

[nph71546-bib-0096] Yachi S , Loreau M . 1999. Biodiversity and ecosystem productivity in a fluctuating environment: The insurance hypothesis. Proceedings of the National Academy of Sciences, USA 96: 1463–1468. 10.1073/pnas.96.4.1463 PMC15485 9990046

[nph71546-bib-0097] Yan C , Zhang Z . 2018. Dome‐shaped transition between positive and negative interactions maintains higher persistence and biomass in more complex ecological networks. Ecological Modelling 370: 14–21.

[nph71546-bib-0098] Yang Z , Shen Y , Li J , Jiang H , Zhao L . 2022. Unsupervised monitoring of vegetation in a surface coal mining region based on NDVI time series. Environmental Science and Pollution Research 29: 26539–26548. 10.1007/s11356-021-17696-9 34854008

[nph71546-bib-0099] Zandalinas SI , Mittler R . 2022. Plant responses to multifactorial stress combination. New Phytologist 234: 1161–1167. 10.1111/nph.18087 35278228

[nph71546-bib-0100] Zhang Z , Yan C , Krebs CJ , Stenseth NC . 2015. Ecological non‐monotonicity and its effects on complexity and stability of populations, communities and ecosystems. Ecological Modelling 312: 374–384.

[nph71546-bib-0101] Zieschank V , Junker RR . 2023. Digital whole‐community phenotyping: tracking morphological and physiological responses of plant communities to environmental changes in the field. Frontiers in Plant Science 14: 1141554. 10.3389/fpls.2023.1141554 PMC10203609 37229120

[nph71546-bib-0102] Zieschank V , Muola A , Janssen S , Lach A , Junker RR . 2025. Tolerance to land‐use changes through natural modulations of the plant microbiome. The ISME Journal 19: wraf010. 10.1093/ismejo/wraf010 PMC11833322 39836381

