# Supplementary material for: Response diversity increases functional but decreases compositional stability of grassland communities
Source: New Phytol. 2026 Sep 6;252(3):1083–95. doi: 10.1111/nph.71546 (PMC13628521; doi:10.1111/nph.71546)
Supplement: Supplementary file 1 [file NPH-252-1083-s001.docx]

## *New Phytologist* Supporting Information

Article title: Response diversity increases functional but decreases compositional stability of grassland communities

Authors: Manuel Pitzer, Vincent Zieschank, Wolfgang Trutschnig and Robert R. Junker* *Corresponding author. Email: robert.junker@uni-marburg.de

Article acceptance date: 28 July 2026

Datasets and R-Code can be found at figshare: https://doi.org/10.6084/m9.figshare.32187945

The following Supporting Information is available for this article:

**Methods S1** Equation for the calculation of the standardized land-use intensity index LUI (historical LUI) which was further used to calculate ∆LUI

**Methods S2** Equation for the calculation of the standardized land-use intensity index LUI used to create abundance-environment relationships (2008 - 2022)

**Fig. S1** Detailed experimental setup of the common garden

**Fig. S2** Comparison of species responses to land-use intensification in field and common garden

**Fig. S3** Community responses to land-use changes in the field and common garden plots as a function of response diversity *RD_2020_*

**Table. S1** Structural Equation Model fit parameters

**Table S2** Regression estimates for Structural Equation Model

**Table S3** ANOVA results of all tested linear mixed-effect models

**Notes S1** A detailed description of the R-package *qad* and a simulation which verifies the suitability *qad* to quantify response dissimilarities and response diversity

$${Lui}_{n_{2008-2019}}=\sqrt{\frac{1}{12}\sum_{t = 2008}^{2019} \left( \frac{F_{n t}}{\overline{F_{2008-2019}}} \right)+ \frac{1}{12} \sum_{t = 2008}^{2019} \left( \frac{M_{n t}}{\overline{M_{2008-2019}}} \right)+ \frac{1}{12} \sum_{t= 2008}^{2019} \left( \frac{G_{n t}}{\overline{G_{2008-2019}}} \right)}$$

**Methods. S1** **Equation for the calculation of the standardized land-use intensity index LUI (historical LUI) which was further used to calculate ∆_LUI_.** Historical LUI integrates three components of land use: the amount of applied fertilizer (**F** in kg N ha-1 year-1), grazing intensity (**G** in livestock units days-1 ha-1 year-1) and mowing frequency (**M** in number of cuts year-1) over the years 2008 to 2019 (the year of grass sod transplantation). The index *n* refers to the individual plot and *t* denotes the year. Therefore, LUI was calculated for each plot *n* as the sum of the averaged, annual mean intensities standardized by the overall mean across years and plot. To reduce the impact of outliers, all values are square-root transformed.

$${Lui}_{n_{2008-2022}}=\sqrt{\frac{1}{15}\sum_{t = 2008}^{2022} \left( \frac{F_{n t}}{\overline{F_{2008-2022}}} \right)+ \frac{1}{15} \sum_{t = 2008}^{2022} \left( \frac{M_{n t}}{\overline{M_{2008-2022}}} \right)+ \frac{1}{15} \sum_{t= 2008}^{2022} \left( \frac{G_{n t}}{\overline{G_{2008-2022}}} \right)}$$

**Methods. S2 Equation for the calculation of the standardized land-use intensity index LUI used to create abundance-environment relationships (2008 - 2022).** LUI integrates three components of land use: the amount of applied fertilizer (**F** in kg N ha-1 year-1), grazing intensity (**G** in livestock units days-1 ha-1 year-1) and mowing frequency (**M** in number of cuts year-1) over the years 2008 to 2022. The index *n* refers to the individual plot and *t* denotes the year. Therefore, LUI was calculated for each plot *n* as the sum of the averaged, annual mean intensities standardized by the overall mean across years and plot. To reduce the impact of outliers, all values are square-root transformed.

**Fig. S1 Detailed experimental setup of the common garden.** The common garden consisted of in total 168 subplots. 156 of those subplots originated from the field plots of the Biodiversity Exploratories and 12 subplots (C) contained bare, steam-sterilized soil at the beginning of our experiment to track spontaneous immigration of species. Per region, 1 m² x 10 cm depth grass sods have been transferred to the Botanical Garden of Marburg, quartered and each of the four parts (= subplots) was assigned to one of the four treatments (HEG 44 represents an example plot from the region Hainich, blue squares indicate that a square of the original grass sod was placed in of the four treatments). Organized in a semi-randomized block design, subplots were covered by a foil belowground and approximately 20 cm apart from each other. Blocks were approximately 150 cm apart from each other. Each of the four subplots per original plot was assigned to one of four experimental treatments: ‘00’ (mowing once per year), ‘0M’ (mowing twice per year), ‘F0’ (mowing once per year and fertilizing) and ‘FM’ (mowing twice per year and fertilizing). Intensity of treatments of common garden subplots (numbers of cuts [cuts / year] and amount of fertilizer [kg N ha^-1^ year^-1^]) were determined based on the 90% quantiles of land-use management data of all plots in the Biodiversity Exploratories between 2006 and 2016 (latest data available at the time of planning) and resulted in LUI values of 00 = 0.99, 0M = 1.40, F0 = 2.07, FM = 2.30. Experimental treatments started in mid-July 2020. Fertilization (99 kg N ha^-1^ year^-1^) and the first mowing of the respective subplots took place in early summer each year, the second mowing took place in autumn.

**Fig. S2** **Comparison of species responses to land-use intensification in field and common garden.** Species responses to land-use intensification in common garden subplots were estimated as the Pearson product-moment correlation coefficient ‘*r*’ by correlating changes in land-se intensity for field to common garden (∆_LUI_) with changes in species abundance from 2020 to 2022 in subplots. We estimated responses for all species the occurred on at least six plots (*n* = 73 of all 96 species). Matching with the previously identified response types (winners, losers, and non-monotonic responders) from field, the species-specific responses observed in the common garden mirrored those recorded in the field (ANOVA: F_2,70_ = 5.701, *p* = 0.0051). Boxplots show the median (central line), interquartile range (box) and whiskers extending to 1.5 interquartile range. Points beyond represent outliers.

**Fig. S3 Community responses to land-use changes in the field and the experimental plots as a function of response diversity *RD_2020_*. (a)** Negative relationship between the historical land-use intensity (LUI) and the response diversity *RD_2020_* of communities in the field (slope = -0.045 (95% Confidence Intervals: -0.083 – -0.008)). **(b)** Changes in Shannon diversity (∆_Shannon_) were negatively related to initial response diversity *RD_2020_* (slope = -2.847 (95% Confidence Intervals: -3.850 – -1.843)). Plant communities with high initial response diversities decreased in Shannon diversity in the course of the experiment and thus were less stable in species diversity. **(c)** Plant communities with high initial response diversities *RD_2020_* had smaller absolute changes in NDVI (|∆_NDVI_|) (slope = -0.159 (95% Confidence Intervals: -0.264 – -0.053)) and were thus functionally more stable than plant communities with low initial response diversity *RD*. **(d)** Negative relationship between experimental changes in land-use intensity (∆_LUI_) and changes in the response diversity (∆*_RD_*) in the course of the common garden experiment (slope = -0.021 (95% Confidence Intervals: -0.038 – -0.004)). All models were tested with linear-mixed effect models containing plot origin as random factor and all model parameters and *p*-values can also be found in Notes S1 Table S3.

**Table S1** Structural Equation Model fit parameters

| **Parameter** | **Value** |
| --- | --- |
| *p*_chi-square_ | 0.59 |
| CFI | 1 |
| TLI | 1.065 |
| RMSEA | 0 |
| SRMR | 0.009 |

**Table S2** Regression estimates for Structural Equation Model. Model was estimated by maximum likelihood estimation. Parameters were bootstrapped 9999 times. Significant terms are indicated by **bold** p-values.

| **Type** | **Dependent variable** | **Predictor variable** | **Estimate** | **SE** | **z** | **p** | **95% CI** | **Standardized estimates** |
| --- | --- | --- | --- | --- | --- | --- | --- | --- |
| Regression | Response Diversity RD_2020_ | LUI | -0.047 | 0.011 | -4.308 | < .001 | -0.068 –  -0.026 | **-0.331** |
| Regression | Inverse\|∆_NDVI_\| | Response Diversity RD_2020_ | 0.163 | 0.044 | 3.696 | < .001 | 0.074 –  0.248 | **0.279** |
| Regression | Inverse\|∆_NDVI_\| | LUI | -0.012 | 0.010 | -1.277 | 0.202 | -0.031 –  0.007 | -0.149 |
| Regression | Inverse\|∆_NDVI_\| | ∆_LUI_ | -0.001 | 0.007 | -0.114 | 0.909 | -0.014 –  0.012 | -0.013 |
| Regression | ∆_Shannon Diversity_ | Response Diversity RD_2020_ | -3.220 | 0.344 | -9.363 | < .001 | -3.861 –  -2.517 | **-0.537** |
| Regression | ∆_Shannon Diversity_ | LUI | -0.210 | 0.087 | -2.405 | 0.016 | -0.384 –  -0.041 | **-0.248** |
| Regression | ∆_Shannon Diversity_ | ∆_LUI_ | -0.200 | 0.062 | -3.207 | 0.001 | -0.322 –  -0.078 | **-0.321** |
| Variance | Initial Response Diversity | Response Diversity RD_2020_ | 0.006 | 0.001 | 9.463 | < .001 | 0.005 –  0.007 | **0.89** |
| Variance | Inverse\|∆_NDVI_\| | Inverse\|∆_NDVI_\| | 0.002 | 0.000 | 10.278 | < .001 | 0.002 –  0.002 | **0.877** |
| Variance | ∆_Shannon Diversity_ | ∆_Shannon Diversity_ | 0.159 | 0.016 | 9.197 | < .001 | 0.123 –  0.190 | **0.669** |
| Co-variance | Inverse\|∆_NDVI_\| | ∆_Shannon Diversity_ | -0.003 | 0.001 | -2.578 | 0.01 | -0.006 –  -0.001 | **-0.193** |
| Co-variance | LUI | ∆_LUI_ | -0.334 | — | — | — | — | -0.741 |
| Indirect  effect | Inverse\|∆_NDVI_\| | LUI *via* Response Diversity RD_2020_ | -0.008 | 0.003 | -2.599 | < .001 | -0.014 – -0.003 | **-0.092** |
| Indirect effect | ∆_Shannon Diversity_ | LUI *via* Response Diversity RD_2020_ | 0.150 | 0.041 | 3.625 | < .001 | 0.076 – 0.238 | **0.178** |

**Table S3** ANOVA results of all tested linear mixed-effect models. All models were tested via sequential ANOVA Type I. Significant terms are indicated by **bold** p-values. *Italic* p-values indicate marginal significance.

| **Figure** | **Explained Variable** | **Term** | **Sum of squares** | **DenDF** | **F-value** | **p(>F)** |
| --- | --- | --- | --- | --- | --- | --- |
| S1.2A | Response Diversity | LUI | 0.0080 | 37.000 | 5.55 | **0.024** |
| S1.2B | ∆_Shannon Diversity_ | ∆_LUI_ | 2.9442 | 147.247 | 25.67 | **< .001** |
|  |  | Response Diversity | 3.5087 | 87.656 | 30.59 | **< .001** |
| S1.2C | \|∆_NDVI_\| | ∆_LUI_ | 0.0051 | 130.694 | 2.96 | *0.088* |
|  |  | Response Diversity | 0.0150 | 69.533 | 8.78 | **0.004** |
| S1.2D | ∆_RD_ | ∆_LUI_ | 0.0286 | 147.998 | 5.77 | **0.018** |
| 5A | Nestedness | ∆LUI | 0.0254 | 141.464 | 1.34 | 0.249 |
|  |  | Response Diversity | 0.1744 | 80.591 | 9.21 | **0.003** |
| 5B | Turnover | ∆_LUI_ | 0.0469 | 143.822 | 6.81 | **0.010** |
|  |  | Response Diversity | 0.0594 | 80.244 | 8.62 | **0.004** |
| - | Sørensen | ∆_LUI_ | 0.1251 | 150.992 | 8.32 | **0.005** |
|  |  | Response Diversity | 0.0333 | 97.627 | 2.21 | 0.140 |
| - | Bray-Curtis Distances | ∆_LUI_ | 0.0365 | 151.058 | 2.24 | 0.137 |
|  |  | Response Diversity | 0.0620 | 98.023 | 3.80 | *0.054* |
| - | Shannon Diversity | LUI | 0.1804 | 37.000 | 4.04 | *0.052* |

**Notes S1**

**Using the R package *qad* to quantify response dissimilarity and response diversity.** Various methods have been introduced to quantify response dissimilarity and response diversity. For instance, response trait diversity has been used as a proxy for response diversity (Craven *et al.*, 2016; Aquilué *et al.*, 2020), which has, however, been criticized for the difficulty in discriminating response from effect traits leading to uncertainties in the interpretation of results (Mori *et al.*, 2013; Ross *et al.*, 2023). More promising may be ‘performance-environment relationships’ (Ross et al. 2023) that directly reflect species responses to environmental changes. Based on performance proxies (such as abundance in studies on pollinators (Winfree & Kremen, 2009; Stavert *et al.*, 2017) or the intrinsic rate of increase in studies on protists and plants (Leary & Petchey, 2009; McCann, 2016; Ross *et al.*, 2023)) of several species along the same environmental gradient, previous studies frequently considered the interaction term *species x environment* as indicative for response diversity. Alternatively, a direct comparison of performance curves of species pairs is a promising approach that reveals more detailed information on species’ responses when using abundances of species (performance proxy) along an environmental gradient (e.g., land-use intensity).

Accordingly, we exploited performance-environment relationships as species-specific responses to an environmental gradient, using abundances of plant species in communities along a gradient of land-use intensity as performance proxy (compare to Ruiz-Moreno *et al.,* 2024; Stavert *et al*., 2017) as observed at *n* = 150 grassland plots within the Biodiversity Exploratories (Fischer *et al.*, 2010) covering three regions in Germany. We modeled the dependence between the performance proxy (abundance) and the environmental gradient (land-use intensity) with empirical checkerboard copulas which are two-dimensional distribution functions that capture full information of, in our case, species-specific abundance-environment relationships (Nelsen, 2007; Durante & Sempi, 2015). Precisely, an empirical checkerboard copula estimates the dependence structure between two variables non-parametrically by dividing the unit square into a grid of equal-sized cells (checkerboard) and assigning density to each, based on observed data ranks. Compared to other approaches, no function, slopes, intercepts or other parameters need to be estimated making them more comparable because of the standardized margins (Junker *et al.*, 2021; Griessenberger *et al.*, 2022a). Additionally, copulas are not restricted to monotonic function types and thus consider non-monotonic relationships between plant abundance and the environment, which are commonly found in natural systems (Hanusch *et al.*, 2023). The dissimilarity between two empirical checkerboard copulas (i.e., between two performance-environment relationships) can be precisely quantified and used as a measure for response dissimilarity *rd_ij_* $\in\left[ 0, 1 \right]$ between species *i* and *j*. A value = 0 indicates a complete similarity, 1 indicates complete dissimilarity. Furthermore, response dissimilarities *rd_ij_* between species in a community can be used to quantify response diversity *RD* of communities analogously to distance-based functional diversity measures (Villéger *et al.*, 2008; Laliberté & Legendre, 2010; Mouchet *et al.*, 2010). In this study, we first generated a response dissimilarity matrix containing pairwise response dissimilarities *rd_ij_* between *n* = 96 plant species frequently found in the plots of the Biodiversity Exploratories where plant species diversity and composition have been recorded since 2008 (Fischer *et al.*, 2010).

For each pair of species, we calculated the dissimilarity of the species-specific abundance-environment curves, which serves as a measure for response dissimilarity *rd_ij_* to LUI, using a copula-based approach as implemented in the R package *qad* (Griessenberger *et al.*, 2022a; Kasper *et al.*, 2022). Therefore, we firstly calculated the mass distribution of the empirical checkerboard copulas for all given plant species with its rolling mean abundances as dependent variable and the mean land-use intensities as the explanatory variable (both based on field data) resulting in *n* = 96 empirical copulas with a resolution of 12. As implemented in the R package *qad*, the resolution was defined as the floor function of the square root of the sample size (*n* = 150 plots), which is the mathematically proven most robust choice for setting the resolution (Junker *et al.*, 2021). Afterwards, *D1* distances between each pair of empirical copulas were calculated, appropriately normalized with the constant 2 to yield response dissimilarity *rd_ij_* $\in[0,1]$ (with 0 meaning identical and 1 indicating maximally different responses) (Trutschnig, 2011; Junker *et al.*, 2021; Griessenberger *et al.*, 2022a,b) and afterwards compiled into a dissimilarity matrix. Our previous publications on *qad* (Junker *et al.*, 2021; Griessenberger *et al.*, 2022a,b) did not mention response dissimilarity but dependence measures. In these papers, we proved the suitability of *qad* to measure the dependence of bivariate datasets. As a matter of fact, measuring the dependence between two variables is a specific version of testing the dissimilarity of two copulas. In the case of measuring dependence, the observed copula (containing the complete information on the dependence of two variables) is compared to a copula representing complete independence. D1 is a generalized measure of the differences between two copulas. Thus, if *qad* as a dependence measure is well performing (as demonstrated in the three peer-reviewed papers mentioned above), it is equally well performing in quantifying the difference in two observed copulas. Nonetheless, using simulated data, we demonstrated that our approach is behaving as expected and thus represents a well-suited method to quantify response dissimilarity and response diversity.

**Simulations**

We designed the simulation to test three expectations that would characterize a well performing index to quantify response dissimilarity. The R script to test these expectations can be found at figshare: <https://doi.org/10.6084/m9.figshare.32187945>

**Expectation 1:** The composition of different responses to the environment determines response diversity. The response diversity *RD* is highest if communities are composed of species that either respond positive or negative; it is lowest if communities are composed of species that have more similar responses to the environment (e.g., positive and non-monotonic). Communities composed of species with positive, negative, and non-monotonic responses to the environment receive response diversity values in between.

**Expectation 2:** The copula-based method to quantify response dissimilarity *rd_ij_* and response diversity *RD* is sensitive to noise in the data. This means that more noisy data result in less response diverse communities (responses become more similar in noisy data sets).

**Expectation 3:** Right-skewed non-monotonic responses become more similar to positive responses, left-skewed non-monotonic responses become more similar to negative responses. These changes in similarity will affect response diversity of communities.

**Results of simulations**

**Expectation 1:** As expected, response diversity *RD* is highest in a community consisting of positively (green) and negatively responding species (yellow, Fig. A). It is lower in communities consisting or either positively or negatively responding species and species that non-monotonically respond to the environment (blue). Communities consisting of species displaying all responses (positive, negative, non-monotonic) received intermediate values.

**Fig. A** Response diversity *RD* of communities with different combinations of species responding either positively (green), negatively (yellow), or non-monotonically (blue) to the environment.

**Expectation 2:** As expected, response diversity *RD* decreased with increasing noise in the data (Fig. B) independent of the composition of the community.

**Fig. B** Response diversity RD as a function of noise in the data. *RD* decreased with increasing noise independent of the composition of the communities, i.e., whether species responding either positively (green), negatively (yellow), or non-monotonically (blue) to the environment. Colored circles (green, yellow, blue) indicate the composition of the simulated communities. Insets in the large figure show examples of no noise, intermediate noise, and strong noise.

**Expectation 3:** As expected, response diversity *RD* reflects species-specific responses to the environment, here modulated by different strength of skewness (Fig. C). Right-skewed non-monotonic responses become more similar to positive responses, left-skewed non-monotonic responses become more similar to negative responses. These changes in similarity affected response diversity of communities.

**Fig. C** Response diversity *RD* as a function of skewness of the non-monotonically responding species. *RD* reflected the similarity of positively (blue), negatively (yellow), or non-monotonically (blue) responding species to another non-monotonically responding species with skewness values ranging from -1 and 1. Colored circles (green, yellow, blue) indicate the composition of the simulated communities. Insets in the large figure show examples of skewness.

**References**

**Aquilué N, Filotas É, Craven D, Fortin M, Brotons L, Messier C**. **2020**. Evaluating forest resilience to global threats using functional response traits and network properties. *Ecological Applications* **30**: e02095.

**Craven D, Filotas E, Angers VA, Messier C**. **2016**. Evaluating resilience of tree communities in fragmented landscapes: linking functional response diversity with landscape connectivity. *Diversity and Distributions* **22**: 505–518.

**Durante F, Sempi C**. **2015**. *Principles of Copula Theory*. New York: Chapman and Hall/CRC.

**Fischer M, Bossdorf O, Gockel S, Hänsel F, Hemp A, Hessenmöller D, Korte G, Nieschulze J, Pfeiffer S, Prati D, *et al.*** **2010**. Implementing large-scale and long-term functional biodiversity research: The Biodiversity Exploratories. *Basic and Applied Ecology* **11**: 473–485.

**Griessenberger F, Junker RR, Trutschnig W**. **2022a**. On a multivariate copula-based dependence measure and its estimation. *Electronic Journal of Statistics* **16**: 2206–2251.

**Griessenberger F, Trutschnig W, Junker RR**. **2022b**. *qad* : An R‐package to detect asymmetric and directed dependence in bivariate samples. *Methods in Ecology and Evolution* **13**: 2138–2149.

**Hanusch M, He X, Janssen S, Selke J, Trutschnig W, Junker RR**. **2023**. Exploring the Frequency and Distribution of Ecological Non-monotonicity in Associations among Ecosystem Constituents. *Ecosystems* **26**: 1819–1840.

**Junker RR, Griessenberger F, Trutschnig W**. **2021**. Estimating scale-invariant directed dependence of bivariate distributions. *Computational Statistics & Data Analysis* **153**: 107058.

**Kasper T, Griessenberger F, Junker RR, Petzel V, Trutschnig W**. **2022**. qad: Quantification of Asymmetric Dependence.

**Laliberté E, Legendre P**. **2010**. A distance-based framework for measuring functional diversity from multiple traits. *Ecology* **91**: 299–305.

**Leary DJ, Petchey OL**. **2009**. Testing a biological mechanism of the insurance hypothesis in experimental aquatic communities. *Journal of Animal Ecology* **78**: 1143–1151.

**McCann MJ**. **2016**. Response diversity of free-floating plants to nutrient stoichiometry and temperature: growth and resting body formation. *PeerJ* **4**: e1781.

**Mori AS, Furukawa T, Sasaki T**. **2013**. Response diversity determines the resilience of ecosystems to environmental change. *Biological Reviews* **88**: 349–364.

**Mouchet MA, Villéger S, Mason NWH, Mouillot D**. **2010**. Functional diversity measures: an overview of their redundancy and their ability to discriminate community assembly rules. *Functional Ecology* **24**: 867–876.

**Nelsen RB**. **2007**. *An Introduction to Copulas*. Springer Science & Business Media.

**Ross SRP-J, Petchey OL, Sasaki T, Armitage DW**. **2023**. How to measure response diversity. *Methods in Ecology and Evolution* **14**: 1150–1167.

**Ruiz-Moreno A, Emslie MJ, Connolly SR**. **2024**. High response diversity and conspecific density-dependence, not species interactions, drive dynamics of coral reef fish communities. *Ecology Letters* **27**: e14424.

**Stavert JR, Pattemore DE, Gaskett AC, Beggs JR, Bartomeus I**. **2017**. Exotic species enhance response diversity to land-use change but modify functional composition. *Proceedings of the Royal Society B: Biological Sciences* **284**: 20170788.

**Trutschnig W**. **2011**. On a strong metric on the space of copulas and its induced dependence measure. *Journal of Mathematical Analysis and Applications* **384**: 690–705.

**Villéger S, Mason NWH, Mouillot D**. **2008**. New Multidimensional Functional Diversity Indices for a Multifaceted Framework in Functional Ecology. *Ecology* **89**: 2290–2301.

**Winfree R, Kremen C**. **2009**. Are ecosystem services stabilized by differences among species? A test using crop pollination. *Proceedings of the Royal Society B: Biological Sciences* **276**: 229–237.
